# Genome-wide association study identifies novel susceptible loci and evaluation of polygenic risk score for chronic obstructive pulmonary disease in a Taiwanese population

**DOI:** 10.1186/s12864-024-10526-5

**Published:** 2024-06-17

**Authors:** Wei-De Lin, Wen-Ling Liao, Wei-Cheng Chen, Ting-Yuan Liu, Yu-Chia Chen, Fuu-Jen Tsai

**Affiliations:** 1 Department of Medical Research, China Medical University Hospital, Taichung, 404327 Taiwan; 2 School of Post Baccalaureate Chinese Medicine, China Medical University, Taichung, 404333 Taiwan; 3 Graduate Institute of Integrated Medicine, College of Chinese Medicine, China Medical University, Taichung, 404333 Taiwan; 4 Center for Personalized Medicine, China Medical University Hospital, Taichung, 404327 Taiwan; 5 Department of Internal Medicine, Pulmonary and Critical Care Medicine, China Medical University Hospital, Taichung, 404333 Taiwan; 6 Graduate Institute of Biomedical Sciences, China Medical University, Taichung, 404327 Taiwan; 7 Department of Medical Research, Million-Person Precision Medicine Initiative, China Medical University Hospital, Taichung, 404327 Taiwan; 8 School of Chinese Medicine, China Medical University, Taichung, 404333 Taiwan; 9 Division of Genetics and Metabolism, China Medical University Children’s Hospital, Taichung, 404327 Taiwan; 10 Department of Medical Genetics, China Medical University Hospital, Taichung, 404327 Taiwan; 11 Department of Medical Laboratory Science and Biotechnology, Asia University, Taichung, 413305 Taiwan; 12 Department of Medical Research, China Medical University Hospital, No. 2, Yude Road, North District, Taichung, 404327 Taiwan

**Keywords:** Chronic obstructive pulmonary disease, Genome-wide association study, Polygenic risk score, Taiwanese population, Genetic association, Genetic Biobank of China Medical University Hospital

## Abstract

**Background:**

Chronic Obstructive Pulmonary Disease (COPD) describes a group of progressive lung diseases causing breathing difficulties. While COPD development typically involves a complex interplay between genetic and environmental factors, genetics play a role in disease susceptibility. This study used genome-wide association studies (GWAS) and polygenic risk score (PRS) to elucidate the genetic basis for COPD in Taiwanese patients.

**Results:**

GWAS was performed on a Taiwanese COPD case–control cohort with a sample size of 5,442 cases and 17,681 controls. Additionally, the PRS was calculated and assessed in our target groups. GWAS results indicate that although there were no single nucleotide polymorphisms (SNPs) of genome-wide significance, prominent COPD susceptibility loci on or nearby genes such as *WWTR1*, *EXT1*, *INTU*, *MAP3K7CL*, *MAMDC2*, *BZW1/CLK1*, *LINC01197*, *LINC01894*, and *CFAP95* (*C9orf135*) were identified, which had not been reported in previous studies. Thirteen susceptibility loci, such as *CHRNA4*, *AFAP1*, and *DTWD1*, previously reported in other populations were replicated and confirmed to be associated with COPD in Taiwanese populations. The PRS was determined in the target groups using the summary statistics from our base group, yielding an effective association with COPD (odds ratio [OR] 1.09, 95% confidence interval [CI] 1.02–1.17, *p* = 0.011). Furthermore, replication a previous lung function trait PRS model in our target group, showed a significant association of COPD susceptibility with PRS of Forced Expiratory Volume in one second (FEV_1_)/Forced Vital Capacity (FCV) (OR 0.89, 95% CI 0.83–0.95, *p* = 0.001).

**Conclusions:**

Novel COPD-related genes were identified in the studied Taiwanese population. The PRS model, based on COPD or lung function traits, enables disease risk estimation and enhances prediction before suffering. These results offer new perspectives on the genetics of COPD and serve as a basis for future research.

**Supplementary Information:**

The online version contains supplementary material available at 10.1186/s12864-024-10526-5.

## Background

Chronic Obstructive Pulmonary Disease (COPD) describes some of the inflammatory lung diseases that cause breathing difficulties. The two most common conditions that fall under the umbrella of COPD are chronic bronchitis and emphysema. COPD is characterized by airflow obstruction, owing to various factors such as inflammation and damage to the airways and lung tissue [1]. Some of the key risk factors that potentially cause COPD are as follows: (a) Smoking: Cigarette smoking is by far the most significant risk factor for COPD. Harmful chemicals in tobacco smoke can irritate and damage the airways and lung tissues over time. (b) Environmental factors: Prolonged exposure to indoor and outdoor air pollutants, including fumes from burning fuels for cooking and heating, increases the risk of COPD. (c) Occupational exposure: People working in certain industries such as mining, construction, and manufacturing may be exposed to dust, chemicals, and fumes that can contribute to the development of COPD [1, 2]. (d) Genetic factors: While smoking and environmental factors play dominant roles, genetic factors can also increase the susceptibility of some individuals to COPD. Genetic variations affect how the lungs respond to damage and inflammation [3].

COPD typically involves complex interactions between genetic and environmental factors. The genetics underlying this group of disease is complex, with the specific genetic factors contributing to COPD remaining an active area of research. Alpha-1 antitrypsin deficiency (AATD) is a hereditary condition caused by mutations in the *SERPINA1* gene. This deficiency leads to the lack of a protective protein (alpha-1 antitrypsin) in the lungs, making individuals with AATD more susceptible to early onset emphysema and COPD. Individuals with two abnormal alleles on the *SERPINA1* gene (homozygous AATD) have a significantly higher risk of developing severe COPD, particularly if they smoke [4]. Variations in certain growth factor genes such as vascular endothelial growth factor, inflammatory and immune response genes such as tumor necrosis factor-alpha and interleukin-6, mucus production genes such as mucin 5B, have been shown to affect the susceptibility to COPD [5–7]. These affect the growth and repair of blood vessels as well as responses to lung damage and inflammation, and could cause excessive mucus production, with the latter causing airway obstruction and respiratory symptoms. Furthermore, surfactant protein genes such as those encoding surfactant proteins A, B, and D, are important for maintaining lung function and variations in these genes have also been associated with a predisposition to COPD [8].

Genome-wide association study (GWAS) has revolutionized our understanding of the genetic basis of complex diseases. GWAS identifies genetic variants associated with a disease by comparing the genomes of people with and without a particular disease. This information can be used to develop new treatments and prevention strategies [9, 10]. Numerous GWASs have been conducted to investigate the genetic basis of COPD. The COPD Genetic Epidemiology Study (COPDGene) is one of the most prominent and extensive GWASs. Genetic and clinical data were collected from thousands of patients with COPD and healthy controls. This study has identified several genetic variants associated with COPD susceptibility and severity, including those related to inflammation, lung development, and oxidative stress genes [11, 12]. A large multicenter observational study, the Evaluation of COPD Longitudinally to Identify Predictive Surrogate Endpoints (ECLIPSE) conducted a GWAS to identify genetic factors contributing to COPD progression and exacerbations and identified genetic variants associated with lung function decline and the risk of exacerbations in COPD patients [13]. The Subpopulations and Intermediate Outcome Measures in COPD Study (SPIROMICS) is another comprehensive study aimed at uncovering the genetic and environmental factors influencing COPD development and progression. A GWAS within SPIROMICS has identified genetic variations linked to lung function decline, emphysema, and other COPD-related traits [14]. The International COPD Genetics Consortium (ICGC) is a collaborative effort involving researchers from around the world focusing on understanding the genetics of COPD. This consortium conducted a GWAS to identify the genetic risk variants and pathways associated with COPD, including genes involved in lung development, inflammation, and mucin production [15]. The GenKOLS Study (Genetics of Chronic Obstructive Lung Disease Study) was based in Norway and conducted a GWAS to identify genetic factors influencing COPD susceptibility and lung function decline. Specific genetic variants associated with COPD risk have been identified in the Norwegian population [16]. In a recent multi-ancestry GWAS meta-analysis of lung function traits in 580,869 individuals, 1,020 independent association single nucleotide polymorphisms (SNPs) implicating 559 genes were identified. These association study results were used to create a genetic risk score for four lung function traits: Forced Expiratory Volume in 1 s (FEV_1_), Forced Vital Capacity (FVC), FEV_1_/FVC ratio, and peak expiratory flow (PEF), which showed a strong association with COPD across ancestry groups [17]. These studies have significantly improved our understanding of the genetic underpinnings of COPD identifying specific disease-associated genetic variations and gene pathways and shedding light on potential targets for future therapeutic interventions.

COPD is a significant health concern in Taiwan, with a prevalence of 6.1% among adults older than 40 years [18]. Determining the specific risk factors and genetic factors associated with COPD in this population is crucial for effective prevention and treatment strategies. Previous studies of COPD in Taiwan were focused on smoking and environmental risk factors [19–21]. Target genes association with COPD have already been reported [22–24]. A recent global biobank meta-analysis paper performed COPD GWAS in combination with other East Asian population biobank data (including Taiwan Biobank), but without independent GWAS or PRS analysis, nor reports on susceptibility genes within the Taiwanese population [25].

The present study, aimed to use GWAS to understand whether Taiwanese people have special genetic factors in COPD and to construct a genetic risk model. Using a custom-designed TPMv1 SNP array [26] and Taiwanese population data, a GWAS was performed to determine the genes and regulatory pathways involved in COPD. GWAS results were employed to build a polygenic risk score (PRS) model to predict COPD using a genetic approach. In addition, a PRS model established in a previous large study based on four different COPD test traits [17] was applied to our COPD study group to evaluate the risk of COPD in the Taiwanese population. These similar genetic factors could be used to explain the risk of COPD in different populations.

## Methods

### Data collection and informed consent

The Precision Medicine Project of the China Medical University Hospital (CMUH) was initiated in 2018 to collect biospecimens and recruit study participants from patients visiting the CMUH. The recruitment and sample collection procedures were approved by the Research Ethics Committee of China Medical University Hospital, Taichung, Taiwan, in accordance with the standards of the Declaration of Helsinki. All participants signed an informed consent form. Blood samples were collected from each participant and clinical information was collected from the electronic medical records (EMRs) of CMUH between 2003 and 2021, with approval by the Research Ethics Committee of CMUH, Taichung, Taiwan.

For sample collection: participants who were 20 years of age or older and had a medical record of COPD diagnosis (ICD-10-CM Diagnosis Code: J44.0, J44.1, J44.9) were considered as COPD cases, and those who had no record of lung/trachea/bronchus disease, cancer, neoplasm, or cardiovascular diseases and were 20 years of age or older were selected as COPD controls.

### Genotyping, imputation, and genome-wide association study

In the present study, the TPMv1 SNP array (TPMv1, Thermo Fisher Scientific, Inc., Waltham, MA, USA), which was developed by the Academia Sinica and Taiwan Precision Medicine Initiative teams was used for genotyping. This array comprised 714,457 SNPs and was employed according to the manufacturer’s protocol [26–28]. SNP data were analyzed using PLINK 2.0 [29]. Participants and SNPs with missing data were excluded if they fulfilled the respective criteria of 10% missing data per individual (–mind 0.1), 10% missing data per marker (–geno 0.1), or heterozygosity > 5 (–het 5 for samples). Next, monomorphic SNPs with a count of < 10 (–mac 10) and multiallelic SNPs were eliminated. Variants with a Hardy–Weinberg equilibrium *P*-value less than 10^−6^ (–hwe 10^−6^) and a minor allele frequency (MAF) less than 10^−4^ (–maf 0.0001) were also excluded. The following analysis criteria were incorporated into our study methodology: heterozygous outliers exceeding a standard deviation value of 5, principal component analysis (PCA) outliers exceeding an interquartile range (IQR) of 3 (for principal components 1 to 10, PC1-10), and mismatches between genotypic sex and actual sex. We also used the KING-robust kinship estimator18 (PLINK 2.0) to remove duplicate samples from our cohort, ensuring that the genetic data were not affected by inflationary effects. After applying these filters, 508,004 variants successfully passed the quality control. Imputation was performed using Beagle 5.2, and whole-genome sequencing data obtained from Taiwan Biobank was used as reference. The imputed data were further filtered based on the following criteria: an alternate allele dosage < 0.3 and a genotype posterior probability < 0.9 [30, 31]. Following quality control and imputation, 14,064,987 variants were analyzed [27].

### Genome-wide association study

The summary statistics were calculated using PLINK 2.0 [29, 32]. The cases and controls were checked using PLINK identity-by-descent (IBD) to remove the first- and second-degree relatives. The selected cases and controls were matched using the MtchIt method [33]. Using PLINK 2.0 in the logistic mode, a GWAS analysis was performed with COPD as the outcome variable. Age and sex were included as covariates in the logistic regression model to account for the potential confounding effects. To address the population structure, PCA was conducted using the EIGENSTRAT method. Adjustments were made for significant PC (PC1–PC10) associated with COPD, as well as demographic variables included age and sex, when estimating odds ratios (ORs) and 95% confidence intervals. The association results were assessed for significance using *P*-values and effect sizes, and a genome-wide significance threshold (*P* < 5 × 10^–8^) was applied to identify significant associations. The R package, ‘qqman’ was used to generate a Manhattan plot and a quantile–quantile (QQ) plot of *P*-values.

### Polygenic risk scores

The objective of our study was to investigate the genetic variations linked to the development of COPD compared to individuals without lung and cardiovascular conditions. We categorized the participants into a base group and a target group for PRS analysis using random allocation (80%: 20%). The base group consisted of 4,354 cases and 14,145 controls, and the target group consisted of 1,088 cases and 3,536 controls. Allocation into COPD cases and controls was based on clinical annotation.

Individual PRS in the target group was estimated using PRSice-2 software (version 2.3.3 for R) by utilizing the ORs obtained from the GWAS data of the base group [34]. SNPs with a *P*-value < 0.05 were selected from the GWAS results of the base group to ensure a sufficient number of significant variants for constructing the PRS model.

The construction of the PRSs was performed using the “clumping and thresholding” approach in PRSice-2. This algorithm iteratively selected a set of SNPs (*P* < 0.05) to form clumps around the index SNPs. Each clump comprised SNPs located within 250 kb of the index SNP and in linkage disequilibrium with the index SNP, based on pairwise threshold of *r*^*2*^ = 0.1. A candidate PRS was computed using the resultant index SNPs and the corresponding estimated OR coefficients for its effect allele as weights using the "score" procedure in the GWAS of the base group [35].

To replicate the PRS obtained from a previous multi-ethnic’s study [17], the list of “best SNPs” of a four-traits (FEV_1_, FVC, FEV_1_/FVC, and PEF) PRS model and their Beta values were applied to our COPD target group to calculate the PRS score using PRSice-2. A total of 1020 SNPs were reported in the previous PRS model: 223 SNPs for FEV_1_, 251 for FVC, 406 for FEV_1_/FVC, and 140 for PEF (Supplementary Table S1). Due to experimental design limitations, only 633 reported SNPs were present in our SNP dataset. For each trait, there were 142 SNPs (64%) for FEV_1_, 151 (60%) for FVC, 257 (63%) for FEV_1_/FVC, and 83 (59%) for PEF. These SNPs are referred to as “best SNPs” and were subjected to PRS calculation (Supplementary Table S2). The PRS was z-score-normalized for comparison (PRS_Z). The average PRS and its standard deviation (SD) were calculated for the cases and controls. A two-sample *t*-test was performed to determine the statistical significance of the difference in PRS between the patients with COPD and controls in target group. We also combined Shrine’s published “best SNPs” [17] and the ORs obtained from our base group to calculate the PRS score in our target group.

### Statistical analysis

To test the statistical power of GWAS, the model proposed by Skol et al. [36] as implemented in a web-based calculation tool (https://csg.sph.umich.edu/abecasis/cats/gas_power_calculator/index.html) was used. The association annotation between SNPs and genes was performed using the ENSEMBL web tool (https://www.ensembl.org/info/docs/tools/vep/index.html), and only genes within 100 kbp surrounding the adjacent SNP were included. D prime and R squared of linkage disequilibrium were calculated using LDmatrix Tool (https://ldlink.nih.gov/?tab=ldmatrix) with 1000 Genomes Project dataset (source: GRCh38 High Coverage, all populations) as reference. The characteristics of the study participants were described by expressing categorical data as proportions. The frequencies of categorical variables were compared using the chi-square test. PRS was normalized (z-score normalization, PRS_Z) and treated as a continuous variable in the models. A *t*-test was used to calculate the significance of PRS in COPD. Receiver-operating characteristic (ROC) curves were generated to quantify the predictive accuracy of PRS models, and the areas under these ROC curves (AUCs) were calculated to assess the discriminatory abilities of the models. Statistical analyses were performed using SPSS (version 21.0; IBM, Armonk, New York, USA) and Excel (2016; Microsoft, Redmond, Washington, USA). All tests were two-sided. Statistical significance was set to a *P* < 0.05.

## Results

**Fig. 1:**
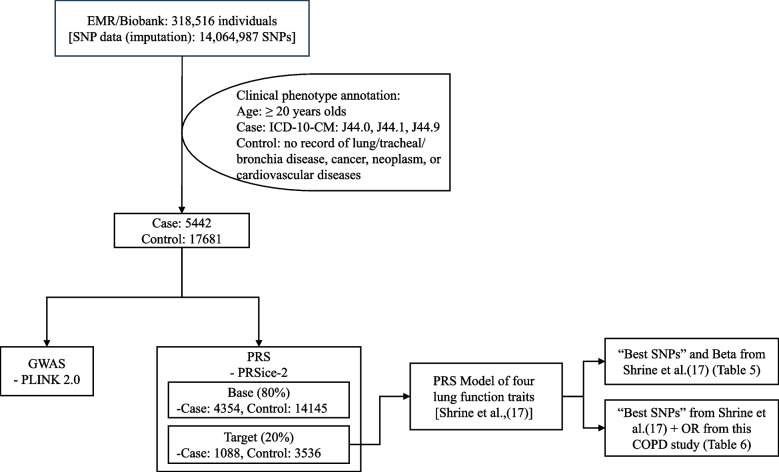
Diagram illustrating the steps involved in electronic medical record (EMR) data mining, genome-wide association study, and polygenic risk score calculation

The complete research process, including EMRs data mining, GWAS, and PRS calculation, is summarized in Fig. 1. After strict quality control procedures, data from 5,442 patients and 17,681 controls were included in the final analysis. The population characteristics of the patients with COPD are shown in Table 1. The mean ages (standard deviation, SD) of the patients and controls were 67.6 (14.7) and 64.3 (14.0) years, respectively. Approximately 69.2% (*N* = 3,766) of patients and 63.0% (*N* = 11,134) of controls were male. A PCA plot of the population structure (PC1 and PC2) is shown in Supplementary Figure S1.

**Table 1:** Selected information on the study population and composition of the base and target groups

	GWAS^a^		PRS^b^			
			Base		Target	
	Case	Control	Case	Control	Case	Control
Number of individuals	5442	17681	4354	14,145	1088	3536
Age (year), mean (SD)	67.6 (14.7)	64.3 (14.0)	67.7 (14.7)	64.3 (14.0)	67.2 (14.8)	64.2 (13.9)
Sex (n, %)						
Male	3766 (69.2)	11,134 (63.0)	3015 (69.2)	8887 (62.8)	751 (69.0)	2247 (63.5)
Female	1676 (30.8)	6547 (37.0)	1339 (30.8)	5258 (37.2)	337 (31.0)	1289 (37.5)

^a^Genome-wide association study

^b^Polygenic risk score

**Fig. 2:**
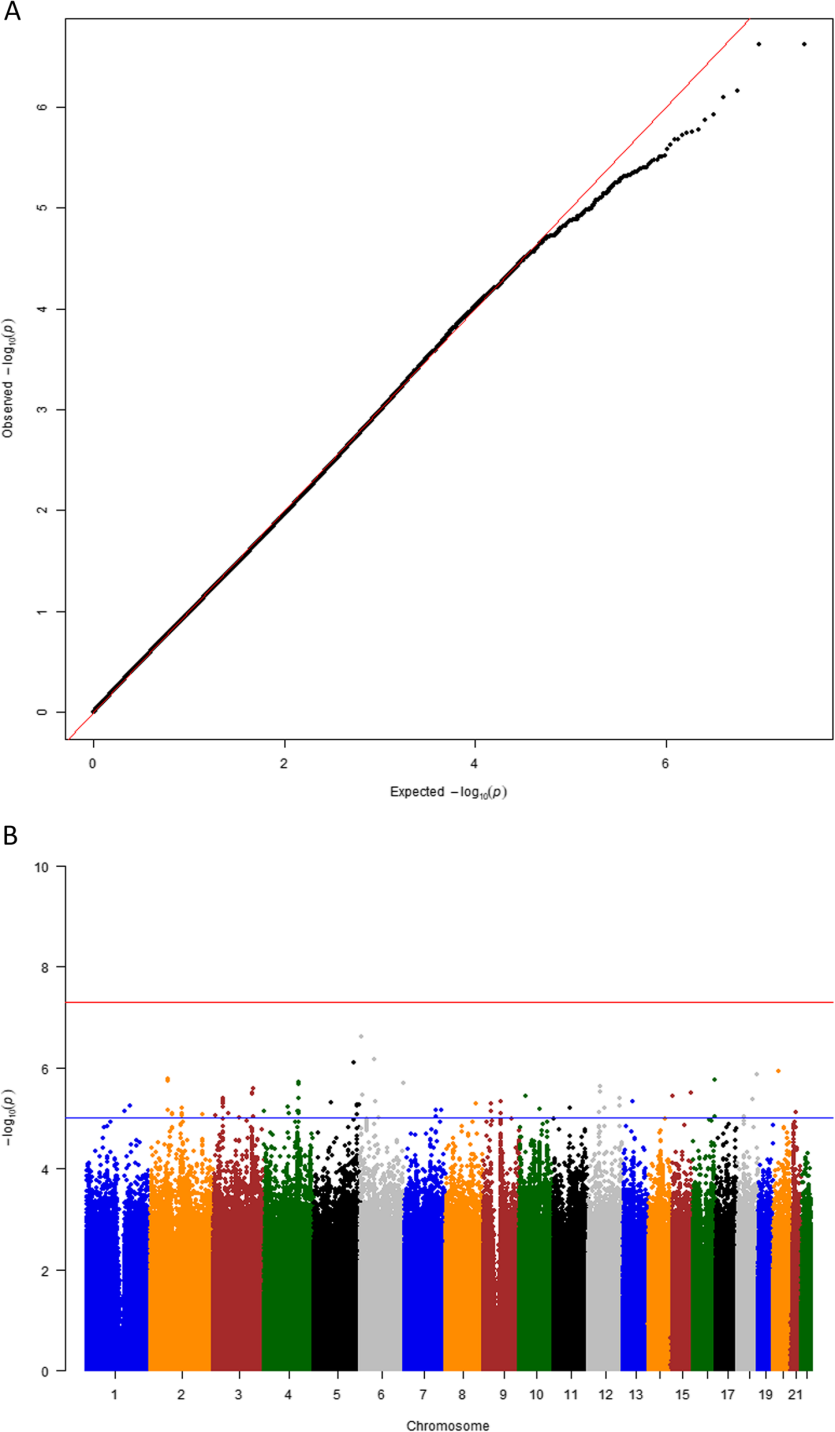
**A** Quantile–quantile plot showing the distribution of observed *P*-values for the identified associations. The plot demonstrates minimal population inflation with a genomic inflation factor (λ) of 1.029. **B** Manhattan plot displaying genome-wide *P*-values for the identified associations. The red line represents the threshold of *P* < 5 × 10^–8^

The QQ plot of SNPs, which compares observed versus expected χ2 test results, did not reveal significant deviation from chance expectations (inflation factor λ = 1.029; Fig. 2A). Although 85 variants exhibited associations with COPD that reached *P* < 1 × 10^−5^ (Fig. 2B, Supplementary Table S3), none reached genome-wide significance (*P* < 5 × 10^−8^). We selected the SNPs with* P* < 1 × 10^−5^ to include SNPs and neighboring genes that showed promising associations with COPD susceptibility. This adjustment allowed us to explore potential relationships with the disease while ensuring a reasonable level of statistical significance. According to the calculation of statistical power using Skol’s model [36], adjustments of MAF and OR were necessary (MAF > 0.05, OR > 1.1) for higher statistical power (0.5 ~ 0.6). The 16 SNPs showing maximum associations when filtered by these conditions are listed in Table 2, marked within genes or adjacent genes (within 100 kbp) following the annotation at the ENSEMBL web tool. The variant with the highest association on chromosome 15p26.2, rs1994147, was found in the *LINC01197* (*LETR1*) region. The other 15 SNPs with maximum association were located in or near the genic region included *WWTR1* on chromosome 3q25.1 (rs6802474/ rs11925206/ rs6783721), *CFAP95* (*C9orf135*) on chromosome 9q21.12 (rs10780705/ rs11140930), *EXT1* on chromosome 8q24.11 (rs12682151), *INTU* on chromosome 4q28.1 (chr4:127564977_G_GT), *MAP3K7CL* on chromosome 21q21.3 (rs57220716), *MAMDC2* on chromosome 9q21.12 (rs10511980), *BZW1/CLK1* on chromosome 2q33.1 (rs2881881/ rs6735908), and a locus in *LINC01894* on chromosome 18q11.2 (rs1786166). Rs58352046, rs76053630, and rs60298813 are located on chromosome 2q14.2. There are no known genes within a distance of 100 kbp. (Supplementary Figure S2).

**Table 2:** The 16 highly associated SNPs identified in the genome-wide association study of COPD

Chr#	Position	rs#	REF	ALT	A1	OR	*P*-value	MAF	Mapped gene	Near gene	Consequence	Source
15	95286295	rs1994147	T	A	A	0.882828	3.13E-06	0.256	*LINC01197* (*LETR1*)		intron	I
3	149699481	rs6802474	T	C	C	0.87027	3.14E-06	0.1775	*WWTR1*		intron	I
3	149690939	rs11925206	A	G	G	0.870656	3.35E-06	0.1776	*WWTR1*		intron	I
3	149692499	rs6783721	C	T	T	0.870656	3.35E-06	0.1776	*WWTR1*		intron	I
9	69924324	rs10780705	C	T	T	1.11071	4.61E-06	0.3886	intergenic	*CFAP95*	downstream	I
8	117746853	rs12682151	C	T	T	0.903904	5.16E-06	0.4961		*EXT1*	intergenic	I
2	118632212	rs58352046	G	A	A	0.818677	6.38E-06	0.07289			intergenic	I
4	127564977	chr4:127564977_G_GT	G	GT	GT	0.880212	7.20E-06	0.2094	intergenic	*INTU*	intergenic	I
21	29161894	rs57220716	C	T	T	0.80475	7.75E-06	0.06402	*MAP3K7CL*		intron	I
9	69929567	rs11140930	C	A	A	1.10816	7.93E-06	0.3697		*CFAP95*	intergenic	I
9	70123423	rs10511980	A	G	A	0.90496	8.02E-06	0.4951	*MAMDC2*		intron	I
2	118625067	rs76053630	G	A	A	0.820916	8.13E-06	0.07303			intergenic	I
2	200840365	rs2881881	T	C	C	0.904296	8.39E-06	0.481		*BZW1/CLK1*	downstream	I
2	200840362	rs6735908	G	A	A	0.904176	8.43E-06	0.48		*BZW1/CLK1*	downstream	I
2	118631503	rs60298813	A	G	G	0.821545	8.72E-06	0.07311			intergenic	I
18	24944932	rs1786166	T	A	A	1.12791	9.54E-06	0.2037	*LINC01894*		intron	I

*Chr#* Chromosome number, *REF* Reference allele, *ALT* Alternative allele, *A1* risk allele, *OR* Odds ratio, *MAF* Minor allele frequency, *Consequence intron* intron variant, *downstream* downstream gene variant, *intergenic* intergenic variant, *Source* Source information of SNP, *G* Genotyped by array, *I* Imputed SNP

Previous GWASs conducted in several different populations identified 1150 susceptibility loci associated with COPD or lung functions (Supplementary Table S4). Hence, these loci were queried in the study population, and the consistent ones with *P* < 0.005, are listed in Table 3 [37–43]. We focused on SNPs with *P* < 0.005 to emphasize high correlations between the datasets without overwhelming complexity. These included several important variants or genes associated with COPD or lung function, such as rs2273500 in *CHRNA4*, rs4488938/rs9654093 in *AFAP1*, rs72731149 in *DTWD1*, rs8070954 in *SMG6*, rs11049488 in *CCDC91*, rs12894780/rs35584079/rs2180369 in *ITPK1*, rs503464 in *CHRNA5*, rs7170068 in *CHRNA3*, rs116921376 in *CYP2F2P*/*CYP2A6*, and rs72927213 in *TUT1*. The findings of other replication analyses with *P*-values larger than 0.005 in our population are presented in Supplementary Table S5.

**Table 3:** Replication analysis of SNPs associated with COPD reported in previous GWAS in the Taiwanese population

										Taiwanese						
Chr	Position	rs#	Allele	Mapped gene	A1	*P*-value	OR	BETA	CI	A1	*P*-value	OR	L95	U95	Trait	Ref
20	63355597	rs2273500	T/C	*CHRNA4*	C	2.00 × 10^–9^	-	0.0947 unit increase	0.064–0.125	C	0.00019	1.123	1.057	1.195	COPD	41
4	7849176	rs4488938	A/G	*AFAP1*	G	1.00 × 10^–8^	-	0.1045 unit increase	0.069–0.14	G	0.00044	1.083	1.036	1.133	COPD	41
4	7902036	rs9654093	G/C	*AFAP1*	G	2.00 × 10^–15^	-	0.0251 unit decrease	0.019–0.031	G	0.00056	0.924	0.883	0.966	COPD	43
15	49692513	rs72731149	G/C	*DTWD1*	G	8.00 × 10^–9^	1.12	-	1.08–1.16	G	0.00073	1.094	1.038	1.152	COPD	37
17	2140495	rs8070954	T/C	*SMG6*	T	5.00 × 10^–13^	-	0.0167 unit decrease	0.012–0.021	C	0.00131	1.074	1.028	1.122	COPD	43
12	28259439	rs11049488	G/A	*CCDC91*	G	5.00 × 10^–8^	1.06	-	1.04 -1.08	G	0.00149	1.230	1.083	1.398	COPD	40
14	93037041	rs12894780	T/C	*ITPK1*	T	4.00 × 10^–20^	-	0.0296 unit decrease	0.023–0.036	C	0.00237	1.131	1.045	1.224	COPD smoker	43
15	78565554	rs503464	T/A	*CHRNA5*	A	8.00 × 10^–11^	0.847	-	0.805–0.890	A	0.00294	0.936	0.896	0.978	COPD	38
15	78620601	rs7170068	G/A	*CHRNA3*	A	2.00 × 10^–9^	0.79	-	0.74–0.86	A	0.00355	0.937	0.897	0.979	COPD smoker	39
14	93045296	rs35584079	G/A	*ITPK1*	A	2.00 × 10^–8^	1.08	-	NR	A	0.00372	1.124	1.039	1.217	COPD	37
14	93050120	rs2180369	T/C	*ITPK1*	C	2.00 × 10^–7^	1.131	-	1.08–1.184	C	0.00441	1.122	1.037	1.215	COPD asthma	42
19	40827821	rs116921376	C/G	*CYP2F2P, CYP2A6*	G	1.00 × 10^–13^	-	0.2402 unit decrease	0.18–0.3	G	0.00449	0.911	0.855	0.972	COPD	41
11	62583726	rs72927213	G/C	*TUT1*	G	2.00 × 10^–18^	-	0.0191 unit increase	0.015–0.023	C	0.00476	0.880	0.806	0.962	COPD	43

*Chr* Chromosome number, *A1* Risk allele, *OR* Odds ratio, *CI* Confidence interval, *L95* Low confidence interval (CI 95%), *U95* High 95% CI, Ref. Reference paper

^$^*P*-values of only those SNPs replicated in our study group (*p* < 0.005) are listed here; the other results are shown in Supplementary Table S5

In this study, 16 SNPs significantly associated with COPD susceptibility were identified. However, the linkage disequilibrium (LD) between these SNPs and previously identified SNPs associated with COPD or lung function traits was found to be low. This indicates that the genetic variants identified in this study may represent novel loci specific to the studied population. The detailed LD relationships, along with the corresponding effect sizes, *P*-values, and MAFs, are summarized in Supplementary Table S6 and Figure S3.

**Fig. 3:**
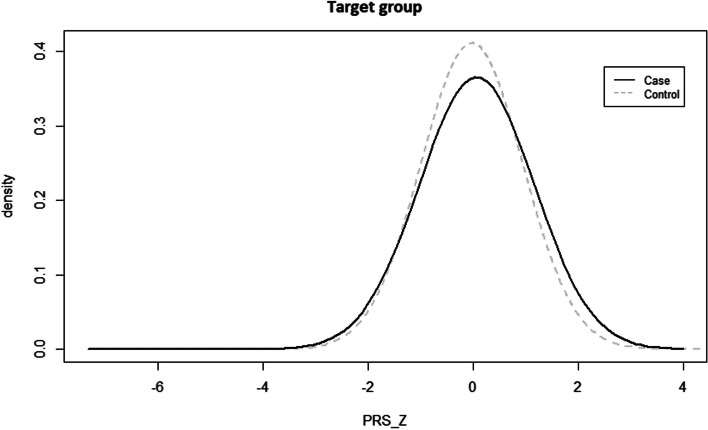
Polygenic risk scoring analysis using the 80% dataset as base and the 20% dataset as target. The *t*-test result of polygenic risk score (Z-score normalization) of COPD cases and controls in target group, *P*-value = 0.011 was statistical significance. PRS_Z, PRS Z-score normalization

The PRS was computed using the summary statistics of the base group and the raw genotypes of the target group using PRSice-2. An optimal SNP combination was derived through iterative calculations. A total of 13,348 SNPs were ultimately selected, with a maximum *P*-value threshold of 0.195 (according to the GWAS of base group). The PRS based on the selected SNPs was calculated for each participant (Supplementary Table S7). A *t*-test was used to test the explanatory capabilities of COPD and PRS_Z (z-score normalization). In the target group, the comparison between cases and controls yielded a *P*-value of 0.011 (*P* < 0.05) (Table 4, Fig. 3), indicating that applying the COPD-PRS model resulted in statistically significant differences.

**Table 4:** *t*-test of PRS_Z for COPD cases and controls in the target group

PRS_Z (Average, SD)					
Control (*n* = 3536)	Case (*n* = 1088)	*df*	*t*	*P*-value	effect size (*d*)
-0.0207 (0.9698)	0.0672 (1.0905)	4622	-2.53	0.011*	0.088

*PRS_Z* Polygenic risk score Z-score normalization, *df* degree of freedom, *t* test statistic, Effect size, Cohen’s d; *P < 0.05

A previously described four-trait PRS model [17] was also applied to the COPD target group. Based on the “best SNPs” and their Beta values, and according to the trait of lung function (FEV_1_/FVC), the average PRS_Z for patients with COPD in the cases of our target group was -0.0918 (SD = 0.9828), while that for controls was 0.0282 (SD = 1.0037). The *t*-test analysis indicated a significant association (*P* = 0.001) between the PRS for FEV_1_/FVC and COPD susceptibility. This suggests that individuals with a higher genetic risk for low FEV_1_/FVC PRS may have an increased genetic predisposition to COPD. The PRS for the other three traits (FEV_1_, FVC, and PEF) did not show any statistical significance in our target group (*P*-values of 0.086, 0.090 and 0.426, respectively) (Table 5).

**Table 5:** *t*-test comparing COPD target group PRS_Zs using published lung function traits PRS models^a^ (best SNPs and Beta)

	PRS_Z (Average, SD)^b^					
Traits	Control (*n* = 3536)	Case (*n* = 1088)	*df*	*t*	*P*-value	effect size (*d*)
Lung function (FEV_1_)	0.0149 (1.0044)	-0.0484 (0.9845)	4622	1.828	0.086	-0.063
Lung function (FVC)	0.0138 (1.0001)	-0.0450 (0.9986)	4622	1.697	0.090	-0.059
Lung function (FEV_1_/FVC)	0.0282 (1.0037)	-0.0918 (0.9828)	4622	3.466	0.001*	-0.120
Peak expiratory flow (PEF)	0.0065 (1.0034)	-0.0211 (0.9892)	4622	0.796	0.426	-0.028

*PRS_Z* Polygenic risk score Z-score normalization, *df* degree of freedom, *t* test statistic, *FEV*_*1*_ Forced expiratory volume, *FVC* Forced vital capacity effect size, Cohen’s d; *P < 0.05

^a^Reference [17]

^b^The selected SNPs (best SNPs) and Beta values for PRS calculation are listed in Supplementary Table S2

Next, the PRS in the target group was calculated for the combined “best SNPs” and the OR values obtained from our analysis of the base group. The averages and SD of PRS_Z for lung function traits are shown in Table 6. With this condition, none of the PRS model of lung function traits reached statistical significance.

**Table 6:** *t*-tests comparing COPD target group PRS_Zs using published SNP list (best SNPs)^a^ and incorporating odds ratios obtained in this study

	PRS_Z (Average, SD)^b^					
Traits	Control (*n* = 3536)	Case (*n* = 1088)	*df*	*t*	*P*-value	effect size (*d*)
Lung function (FEV_1_)	-0.0148 (1.0036)	0.0480 (0.9873)	4622	-1.810	0.070	0.063
Lung function (FVC)	-0.0113 (1.0317)	0.0367 (0.8886)	4622	-1.368	0.166	0.048
Lung function (FEV_1_/FVC)	-0.0149 (1.0062)	0.0485 (0.9785)	4622	-1.830	0.067	0.063
Peak expiratory flow (PEF)	-0.0005 (1.0005)	0.0016 (0.9988)	4622	-0.060	0.952	0.002

*PRS_Z* Polygenic risk score Z-score normalization, *df* degree of freedom, *t* test statistic, *FEV*_*1*_ Forced expiratory volume, *FVC* Forced vital capacity effect size, Cohen’s d; *P < 0.05

^a^Reference [17]

^b^The selected SNPs for PRS calculation are listed in Supplementary Table S2

The trend of a PRS is inherently linked to the trait it aims to assess. In multi ethnics studies, using lung function as the indicator for PRS establishment, lung function values represent health status numerically and higher values denote better lung function. As shown in Table 5, we found that the PRS for controls was higher (indicating better lung function), while that for cases was lower. Conversely, when we based the PRS on the presence or absence of COPD aiming to predict COPD risk, the scenario changed to one where the PRS for cases tended to be higher, signifying a greater risk of COPD, while it was relatively low for control (Table 6). Consequently, evaluation of the two tables must be based on the chosen perspective.

We also investigated the ability of the PRSs to distinguish between individuals with and without COPD. The significance (*P*-value), odds ratio, and the amount of variance explained (R^2^) derived from this analysis are shown in Supplementary Table S8. In the target group, an increase in the PRS was associated with increased COPD risk in the logistic regression model (OR 1.094, 95% CI 1.020–1.172, R^2^ = 0.0021). Of the four examined lung function traits PRS model in the target group, only the FEV_1_/FVC trait calculated as “best SNPs + Beta” showed an improved distinguishing capability (OR 0.886, 95% CI 0.828–0.949). In the target group, the AUC was 0.528 (95% CI 0.508–0.548) and 0.534 (95% CI 0.514–0.553), respectively, for the PRS of the regression models using our study (COPD PRS) and FEV_1_/FVC trait PRS. Other results are shown in Supplementary Figure S4.

## Discussion

Based on the relationship between SNPs and genes, the 16 identified SNPs showing maximum association could be divided into three groups: 1) Intron variant; most SNPs belonged to this group, including rs11925206, rs6783721, rs6802474, rs10511980, rs1994147, rs1786166, and rs57220716. 2) Downstream gene variant; the SNP is located within 20 kbp downstream of adjacent genes, including rs6735908, rs2881881, and rs10780705. 3) Intergenic variant; all other SNPs belonged to this group, including rs76053630, rs60298813, rs58352046, chr4:127564977_G_GT, rs12682151, and rs11140930. The aforementioned 16 SNPs still require further research to confirm their effects on gene expression or regulation. The known genes most strongly associated with these SNPs, within genes or adjacent genes (within 100 kbp), were *WWTR1*, *EXT1*, *MAP3K7CL*, *MAMDC2*, *BZW1/CLK1*, *INTU*, *CFAP95*, *LINC01197* (*LETR1*), and *LINC01894*. These genes were not identified in previous GWAS.

*LINC01197* (*LETR1*) and *LINC01894* are long noncoding RNAs (lncRNAs). Several studies have identified dysregulated expression of lncRNAs in COPD patients compared to healthy individuals. These lncRNAs have been implicated in various cellular processes involved in COPD pathogenesis, such as inflammation, oxidative stress, and airway remodeling. Some lncRNAs have also been proposed as potential biomarkers for COPD diagnosis, prognosis, and treatment response [44–48]. In addition, *LINC01197* (*LETR1*) is a lymphatic endothelium-specific long noncoding RNA governing cell proliferation and migration [49]. However, its significance to respiratory disease, specifically COPD, requires further investigation.

*WWTR1* is involved in various cellular processes including cell proliferation and tissue repair. Variations in *WWTR1* may influence lung tissue repair mechanisms and airway remodeling [50]. In a recent study, downregulation of *WWTR1* was observed in COPD samples compared to healthy samples [51]. This suggests that *WWTR1* gene expression is crucial for normal cellular function. Our results indicate that the SNPs located in *WWTR1* have ORs less than 1 (OR = 0.87), implying a protective effect against COPD. This finding aligns with the higher expression of *WWTR1* in normal cells observed in cell expression analyses. Currently, there are no reports on whether these three intronic SNPs influence the gene expression of *WWTR1*. Further experiments are needed in the future to establish this association. Additionally, *WWTR1* is known to be associated with ferroptosis, a form of programmed cell death induced by lipid peroxidation through an iron-dependent pathway [52–54]. Ferroptosis has been implicated in various lung diseases, including COPD [53–55], highlighting the potential importance of *WWTR1* in COPD pathogenesis. These observations underscore the need for further investigation into the role of *WWTR1* and ferroptosis-related pathways in COPD development and progression.

The *EXT1* gene encodes a glycosyltransferase enzyme called exostosin-1. This enzyme is involved in the biosynthesis of heparan sulfate (HS), a type of polysaccharide that is a component of proteoglycans. Proteoglycans are important for the structure and function of connective tissues, including cartilage and bone. Mutations in the *EXT1* gene can lead to a condition called hereditary multiple exostoses, which is characterized by the formation of benign bone tumors called osteochondromas [56]. In chronic lung diseases like asthma and COPD, macrophages exhibit a phenotype similar to that of alternatively activated (M2) macrophages, characterized by an upregulation of HS biosynthesis genes. However, *EXT1* expression is not significantly regulated in M2-like macrophages from patients with chronic lung diseases, suggesting a different role for *EXT1* under these conditions compared to other diseases like rheumatoid arthritis and atherosclerosis, where *EXT1* expression is increased [57]. In addition, an SNP, rs74701635, located approximately 49 kbp downstream of the *EXT1* gene, has been associated with smoking behavior [58]. This SNP is about 776 bp away from another SNP, rs12682151, which was identified in this study. While the exact functional significance of these SNPs in relation to *EXT1* and COPD remains unclear, their proximity to the *EXT1* gene suggests a potential link between genetic variation in this region and smoking behavior, which is a known risk factor for COPD.

The *MAP3K7CL* gene, also known as MAP3K7 C-terminal like, may be involved in signaling pathways that regulate various cellular processes such as cell growth, differentiation, and apoptosis. In a gene expression study on tumor-educated leukocytes mRNA isolated from non-small cell lung cancer patients, *MAP3K7CL* was found to be downregulated [59]. Research on its specific role in COPD is currently lacking. The *MAMDC2* gene, also known as MAM domain containing 2, is involved in various biological processes, including cell adhesion, migration, and signaling. A study reported that *MAMDC2* exhibited tumor-suppressive activity and may constitute a biomarker for breast cancer treatment [60]. The *BZW1* gene, also known as Basic Leucine Zipper and W2 Domains 1, encodes a protein involved in transcriptional regulation. Abnormal expression of this gene is associated with a variety of cancers [61, 62]. In addition, *BZW1*, as a translation initiation regulation factor, plays an important role in preimplantation embryo protein synthesis [63]. However, its association with COPD remains to be studied. The *CLK1* gene, also known as CDC2-Like Kinase 1, encodes a protein belonging to the CLK family of serine/threonine kinases. These kinases play crucial roles in regulating pre-mRNA splicing, which is essential for the production of mature mRNA transcripts [64]. While *CLK1*'s direct role in lung biology is unclear, its involvement in mRNA splicing suggests an indirect influence on lung function and disease, given the importance of proper splicing for lung health. *INTU* (Inturned Planar Cell Polarity Protein) is associated with embryonic digit and mouth development, functioning in the ciliary basal body and motile cilium. It is linked to conditions like asphyxiating thoracic dystrophy and orofaciodigital syndrome XVII. *INTU* plays a crucial role in ciliogenesis, regulating cilia formation and cell polarity, indirectly impacting Hedgehog signaling. Mutations in *INTU* and related ciliary genes contribute to orofacial-digital syndromes and ciliopathies, highlighting its significance in cilia formation and cellular processes [65, 66]. While its direct association with lung function has not been well established, planar cell polarity pathways may indirectly affect lung development [67]. *CFAP95* (*C9orf135*) encodes a membrane-associated protein that may serve as a surface marker for undifferentiated human embryonic stem cells [68]. The function of the *CFAP95* (*C9orf135*) gene has not been extensively studied, and its specific role in lung biology remains unclear. Further research is needed to determine any potential relevance to the lungs.

In addition to the highly associated genes discovered, our results identified those previously reported as COPD-or lung function-related genes including *CHRNA3*, *CHRNA4*, *CHRNA5*, *AFAP1*, *SMG6*, *ITPK1*, *CYP2A6*, *TUT1*, *DTWD1*, and *CCDC91* in our study cohort. *CHRNA3*, *CHRNA4*, and *CHRNA5* encode the subunits of nicotinic acetylcholine receptors (nAChRs) involved in the neurotransmission of acetylcholine. Variations in these genes render individuals more susceptible to nicotine dependence. Because smoking is a major risk factor for COPD, individuals with these genetic variants are at a higher risk of developing COPD. Furthermore, these genes have been linked to changes in lung function even in patients without COPD. Variations in *CHRNA3* and *CHRNA5* levels are associated with reduced lung function, FEV_1_ and FVC, which may contribute to the development of COPD [69]. *AFAP1* is involved in actin cytoskeleton organization and cell motility. Variations in the gene related to cytoskeletal dynamics can potentially affect airway remodeling and lung function in COPD [70]. *SMG6* is involved in the nonsense-mediated mRNA decay pathway, which is involved in mRNA surveillance and degradation. Variations in the gene involved in mRNA stability and processing may affect the regulation of inflammation and tissue repair in COPD [71]. *ITPK1* is involved in the regulation of inositol phosphate metabolism, which affects cell signaling pathways. Variations in genes involved in intracellular signaling pathways may have downstream effects on inflammatory responses in the lungs [72]. *CYP2A6* is an enzyme responsible for metabolizing nicotine and other tobacco-related compounds. Genetic variants of *CYP2A6* influence an individual's ability to metabolize nicotine, which may in turn affect smoking behavior and susceptibility to COPD [73]. *TUT1* is involved in RNA modification and degradation. Variations in RNA processing genes may influence the stability and regulation of genes associated with lung function and inflammation [74]. *DTWD1* possesses tRNA-uridine aminocarboxypropyltransferase activity and is involved in tRNA modification. Its role in lung function and COPD is not well established, and further research is required to understand its significance in respiratory health. However, the specific role of *CCDC91* in COPD has not been well documented. Genetic variants of this gene may influence processes related to lung function and airway inflammation [75].

Based on the GWAS results, many genes have been previously linked to either COPD or lung function traits, indicating their potential relevance to respiratory health. However, it is important to note that the genetic basis of COPD is multifactorial, and that these genes likely interact with other genetic and environmental factors to contribute to disease susceptibility and severity. Further research is needed to elucidate the specific mechanisms by which these genes influence COPD and lung function.

The results of the GWAS in the Taiwanese COPD study group suggested a significant genetic component of COPD. The PRS analysis using PRSice-2 also supported this finding, showing statistical significance in the target groups. The *t*-test yielded a *P*-value of 0.011 and logistic regression yielded OR 1.09 (95% CI 1.02–1.17) and AUC 0.528 (95% CI 0.508–0.548), suggesting that the identified genetic variants were significantly correlated with COPD.

Furthermore, a previously established PRS model for lung function traits [17] was applied to our target group, which included a set of SNPs associated with lung function traits such as FEV_1_, FVC, FEV_1_/FVC, and PEF. The PRS model of FEV_1_/FVC revealed statistical significance between our COPD cases and controls. The FEV_1_/FVC ratio is used to assess pulmonary mechanical limitations, such as airflow restriction commonly seen in COPD patients. A lower ratio may indicate more impaired lung function. Using the lung function trait, FEV_1_/FVC, to establish a genetic PRS model, higher scores may indicate better lung function and lower chances of developing COPD, leading to a decrease in the odds ratio for risk. Using associations found through GWAS and PRS, there is potential to elucidate the molecular mechanisms underlying changes in lung function, thereby understanding the pathogenesis of COPD at a molecular level. This might include more information about lung function measurements and further explanation of the relationship between the FEV_1_/FVC ratio and COPD. However, the PRS for FEV_1_, FVC, and PEF did not show statistically significant associations with COPD in our target group. Furthermore, when using the “best SNPs” and our ORs to calculate the PRS, no PRS model of the four lung function traits reached statistical significance.

Shrine et al. [17] generated a PRS for four lung function traits based on 49 study cohosts. In these ethnic groups, 80.6% were of European ancestry and 14.7% were of East Asian ancestry, which is closer to our ethnicity. Interestingly, replicating their PRS model to our COPD target group, on the “best SNPs”, could distinguish between cases and controls in a comparable manner to our PRS. This indicates common genetic factors for COPD or lung function traits across ethnic groups. However, based on our GWAS and PRS results, we found that some novel risk variants or loci are associated with COPD.

PRS are often developed based on GWAS conducted on specific populations or ethnic groups. This means that the genetic variants and their effect sizes used to calculate the PRS may be more applicable and accurate within the population from which they were derived. Consequently, the PRS developed in one ethnic group may not perform as well in individuals from different ethnic backgrounds. Historically, many GWAS have been conducted in populations of European ancestry, leading to biases in the available genetic data. Consequently, PRSs developed using these data may not be informative for individuals from non-European ethnic backgrounds. To address this limitation, researchers have attempted to include diverse populations in their genetic studies. Genetic variants associated with certain traits or diseases occur at different frequencies across ethnic groups. Variants common to one population may be rare in another. This can influence the performance of a PRS when applied to individuals from different ethnic backgrounds. The PRS may need to be recalibrated or adapted for specific populations [76, 77].

PRS is a valuable tool for assessing an individual's genetic inclination towards specific diseases, allowing for personalized prevention and screening approaches. Moreover, PRS assists in disease identification, prognosis, and treatment selection, aiding in the identification of suitable candidates for clinical trials based on their genetic risk profiles. It is important to note that PRS analysis relies on statistical associations rather than causation, necessitating further research to validate the connection between genetic variants and understand the underlying biological mechanisms [78].

Overall, current GWAS investigations on COPD have provided valuable insights into the genetic foundations of this intricate condition. Although the identified genetic variants may exert only a modest influence and elucidate only a fraction of the genetic complexity of COPD, they offer valuable insights into the underlying biological processes associated with the disease. The replication findings presented here provide important information regarding lung function traits in the Taiwanese population with meaningful implications for both clinical practice and public health. The susceptibility genes identified in this study may serve as promising targets for future prevention and treatment strategies involving drug development and personalized therapeutic approaches [79, 80]. In this study, PRS demonstrated statistical significance based on genetic information, but future investigations with larger sample sizes have the potential to enhance the identification of highly representative genetic susceptibility loci, enabling the simplification of personalized PRS. This approach can further incorporate both genetic and environmental factors to identify individuals at heightened risk of developing COPD. The capacity for prediction or early diagnosis can guide timely management and intervention.

Our study is subject to some limitations. Firstly, while we acknowledge the influence of factors such as smoking, environmental exposures, socioeconomic status, and disease severity or specific phenotypes on COPD susceptibility, the incomplete records in the EMRs prevented us from including these variables in our analysis. This may have introduced bias into our results, given the established associations between these factors and COPD risk. Additionally, the limited number of cases available for analysis within the timeframe of our study resulted in insufficient statistical power (> 0.8; the necessary sample size would exceed 8000 cases), which may have affected the robustness of our findings. As a result, we were unable to explore potential associations between these factors and COPD susceptibility. To address these limitations, we will continue to collect more comprehensive patient data and collaborate with other medical centers to obtain replication cohorts for further validation in future studies.

## Conclusions

This study performed GWAS and PRS construction using data from a Taiwanese cohort of 5,442 COPD cases and 17,681 non-COPD individuals as controls. Common and novel COPD susceptibility loci were identified and compared with previous GWAS results from different populations. Although no SNP reached the genome-wide significance, we identified *WWTR1*, *EXT1*, *INTU*, *MAP3K7CL*, *MAMDC2*, *BZW1/CLK1*, *LINC01197*, *LINC01894*, and *CFAP95* (*C9orf135*) as prominent COPD susceptibility loci found in Taiwan. Furthermore, replication and confirmation of susceptibility loci between Taiwanese and other populations were achieved. The PRS results obtained in our study group or other population groups could be an effective tool for the quantification of polygenic contributions to COPD at the individual level. Our findings demonstrated a significant association between the PRS and COPD susceptibility in the study population. The established PRS model may serve as a valuable genetic tool for identifying individuals at a higher risk of developing COPD.

### Supplementary Information

Supplementary Material 1.

Supplementary Material 2.

Supplementary Material 3.

Supplementary Material 4.

Supplementary Material 5.

Supplementary Material 6.

## Data Availability

Data supporting the findings of this study are available from the corresponding author upon request. GWAS summary statistics data link: https://my.locuszoom.org/gwas/255056/?token=42c73f97b16c476eb75b23a928aec182
