# Supplementary material for: Genome-wide association study identifies novel susceptible loci and evaluation of polygenic risk score for chronic obstructive pulmonary disease in a Taiwanese population
Source: BMC Genomics. 2024 Jun 17;25:607. doi: 10.1186/s12864-024-10526-5 (PMC11184693; doi:10.1186/s12864-024-10526-5)
Supplement: Supplementary file 2 — Supplementary Material 2. [file 12864_2024_10526_MOESM2_ESM.docx]

Supplementary Table S8. Effect of polygenic risk score (with different models) on COPD risk in the logistic regression model

|  | PRS calculation (Traits) | Significance (*P*-value) | Exp(B) (Odds Ratio, CI 95%) | R^2^ |
| --- | --- | --- | --- | --- |
| PRS Calculation reference | 20_target (this study) | *0.011* | 1.094 (1.020-1.172) | *0.002112* |
| Best SNPs + Beta | FEV1 | 0.068 | 0.939 (0.877-1.005) | 0.001088 |
|  | FVC | 0.090 | 0.943 (0.881-1.009) | 0.000938 |
|  | FEV1/FVC | *0.001* | 0.886 (0.828-0.949) | *0.003906* |
|  | PEF | 0.426 | 0.973 (0.909-1.041) | 0.005771 |
| Best SNPs + OR | FEV1 | 0.070 | 1.065 (0.995-1.140) | 0.001066 |
|  | FVC | 0.164 | 1.053 (0.979-1.132) | 0.000655 |
|  | FEV1/FVC | 0.067 | 1.066 (0.995-1.141) | 0.001091 |
|  | PEF | 0.952 | 1.002 (0.936-1.073) | 0.000001 |

Exp(B), odds ratio; CI 95%, 95% confidence interval; R^2^, explanatory power.
