# Supplementary material for: Genome-wide association study identifies novel susceptible loci and evaluation of polygenic risk score for chronic obstructive pulmonary disease in a Taiwanese population
Source: BMC Genomics. 2024 Jun 17;25:607. doi: 10.1186/s12864-024-10526-5 (PMC11184693; doi:10.1186/s12864-024-10526-5)
Supplement: Supplementary file 3 — Supplementary Material 3. [file 12864_2024_10526_MOESM3_ESM.pptx]

## Slide 1
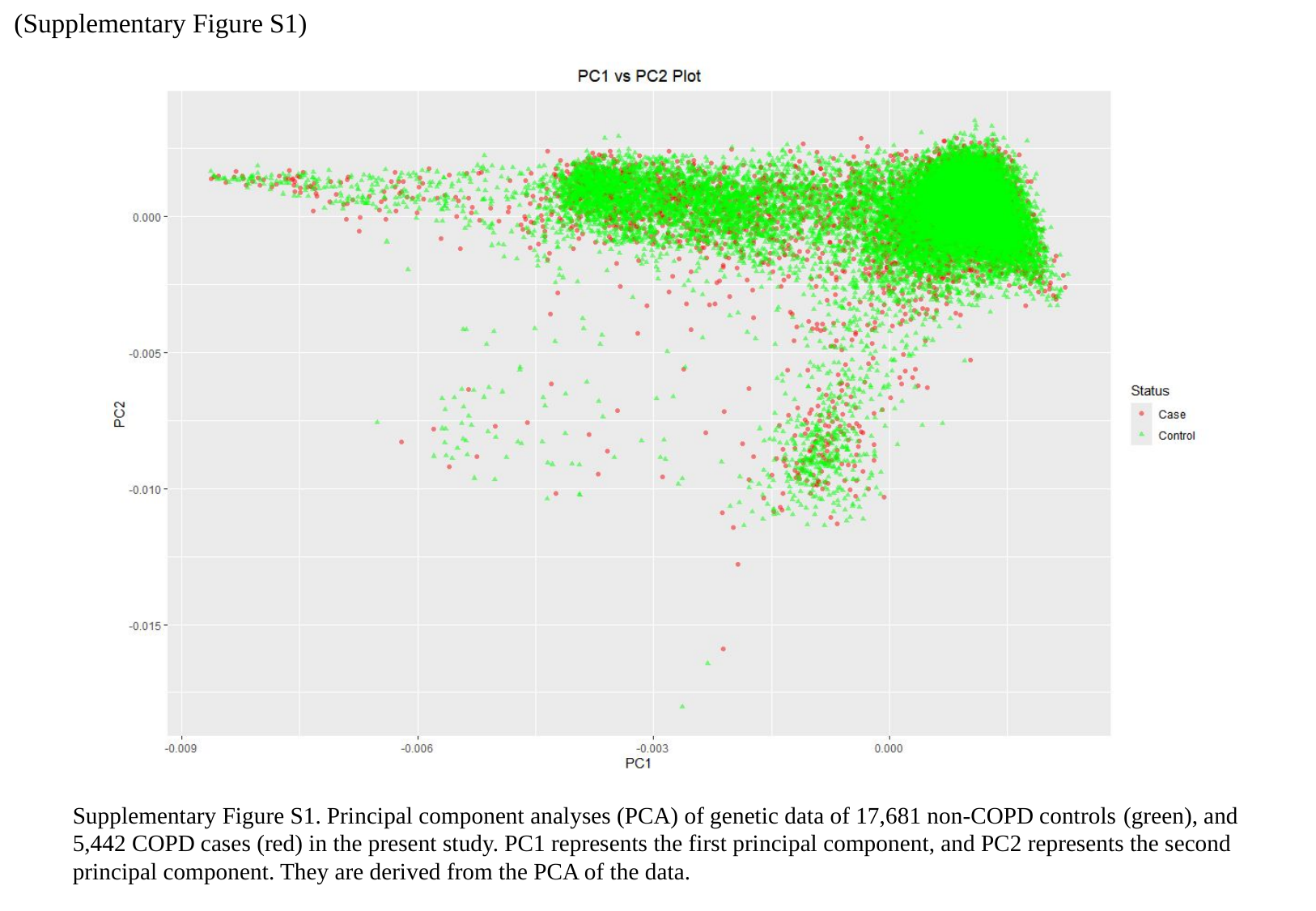

(Supplementary Figure S1)
Supplementary Figure S1. Principal component analyses (PCA) of genetic data of 17,681 non-COPD controls (green), and 5,442 COPD cases (red) in the present study. PC1 represents the first principal component, and PC2 represents the second principal component. They are derived from the PCA of the data.
