# Supplementary material for: Genome-wide association study identifies novel susceptible loci and evaluation of polygenic risk score for chronic obstructive pulmonary disease in a Taiwanese population
Source: BMC Genomics. 2024 Jun 17;25:607. doi: 10.1186/s12864-024-10526-5 (PMC11184693; doi:10.1186/s12864-024-10526-5)
Supplement: Supplementary file 4 — Supplementary Material 4. [file 12864_2024_10526_MOESM4_ESM.pptx]

## Slide 1
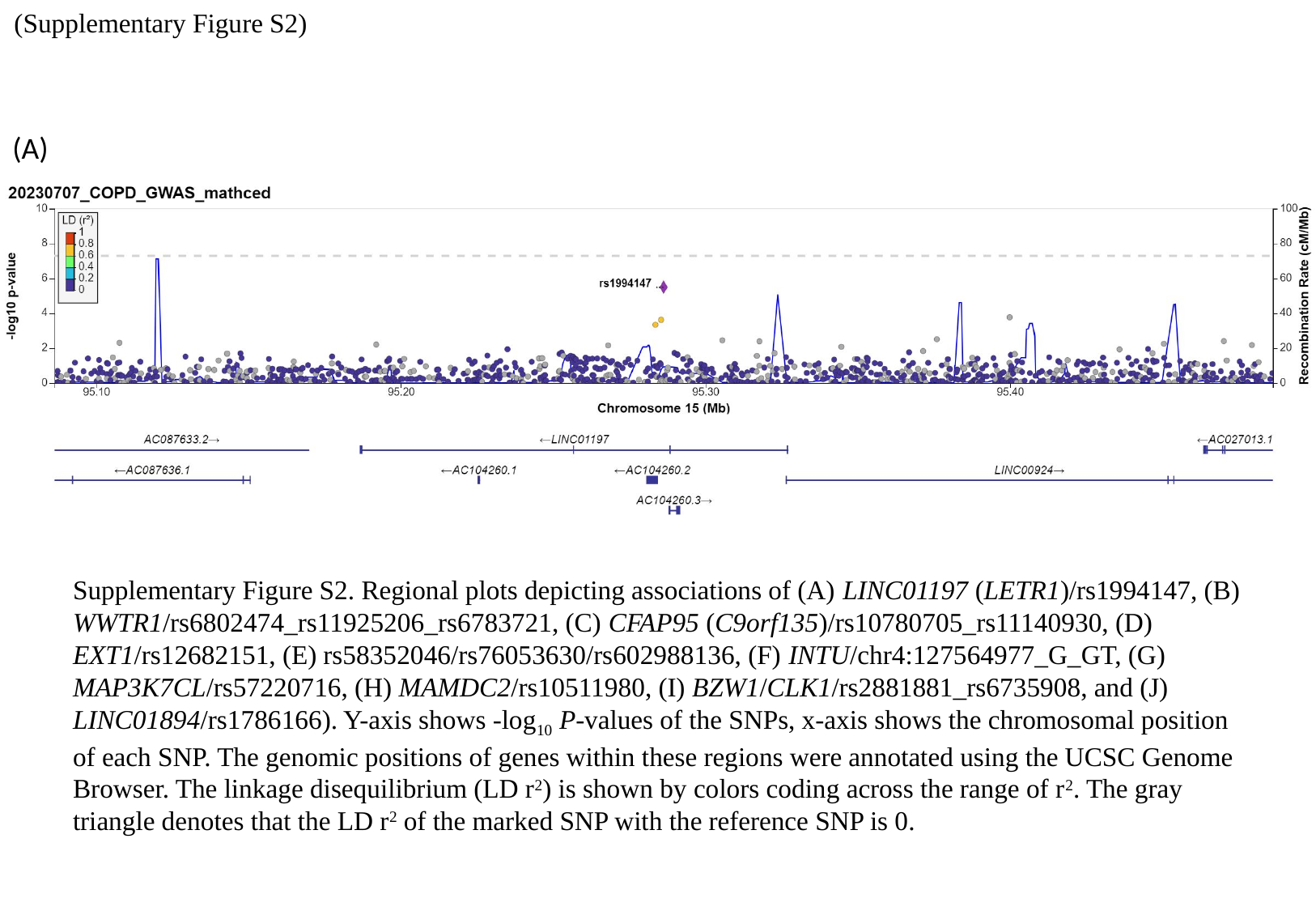

(Supplementary Figure S2)
(A)
Supplementary Figure S2. Regional plots depicting associations of (A) LINC01197 (LETR1)/rs1994147, (B) WWTR1/rs6802474_rs11925206_rs6783721, (C) CFAP95 (C9orf135)/rs10780705_rs11140930, (D) EXT1/rs12682151, (E) rs58352046/rs76053630/rs602988136, (F) INTU/chr4:127564977_G_GT, (G) MAP3K7CL/rs57220716, (H) MAMDC2/rs10511980, (I) BZW1/CLK1/rs2881881_rs6735908, and (J) LINC01894/rs1786166). Y-axis shows -log10 P-values of the SNPs, x-axis shows the chromosomal position of each SNP. The genomic positions of genes within these regions were annotated using the UCSC Genome Browser. The linkage disequilibrium (LD r2) is shown by colors coding across the range of r2. The gray triangle denotes that the LD r2 of the marked SNP with the reference SNP is 0.

## Slide 2
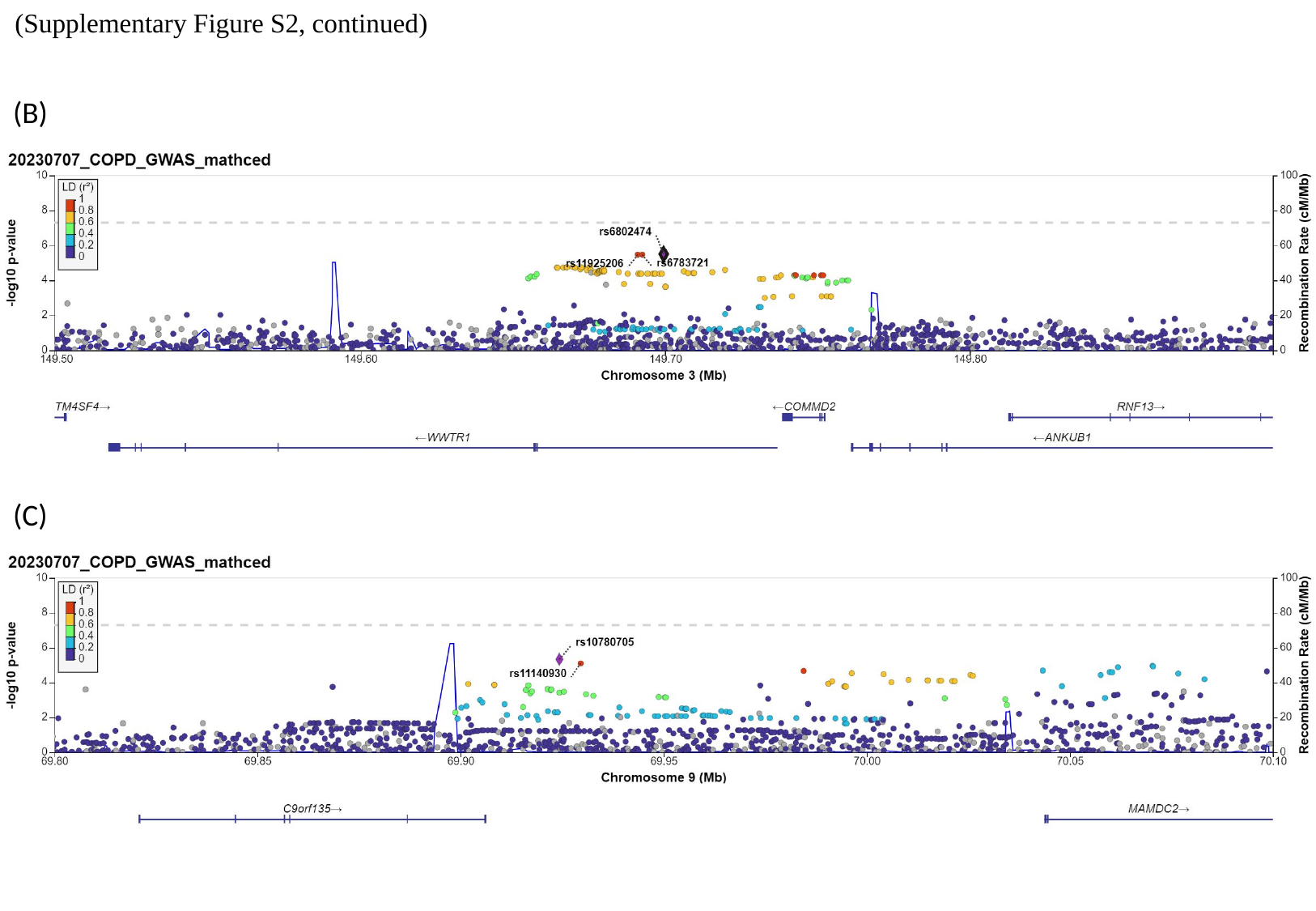

(Supplementary Figure S2, continued)
(B)
(C)

## Slide 3
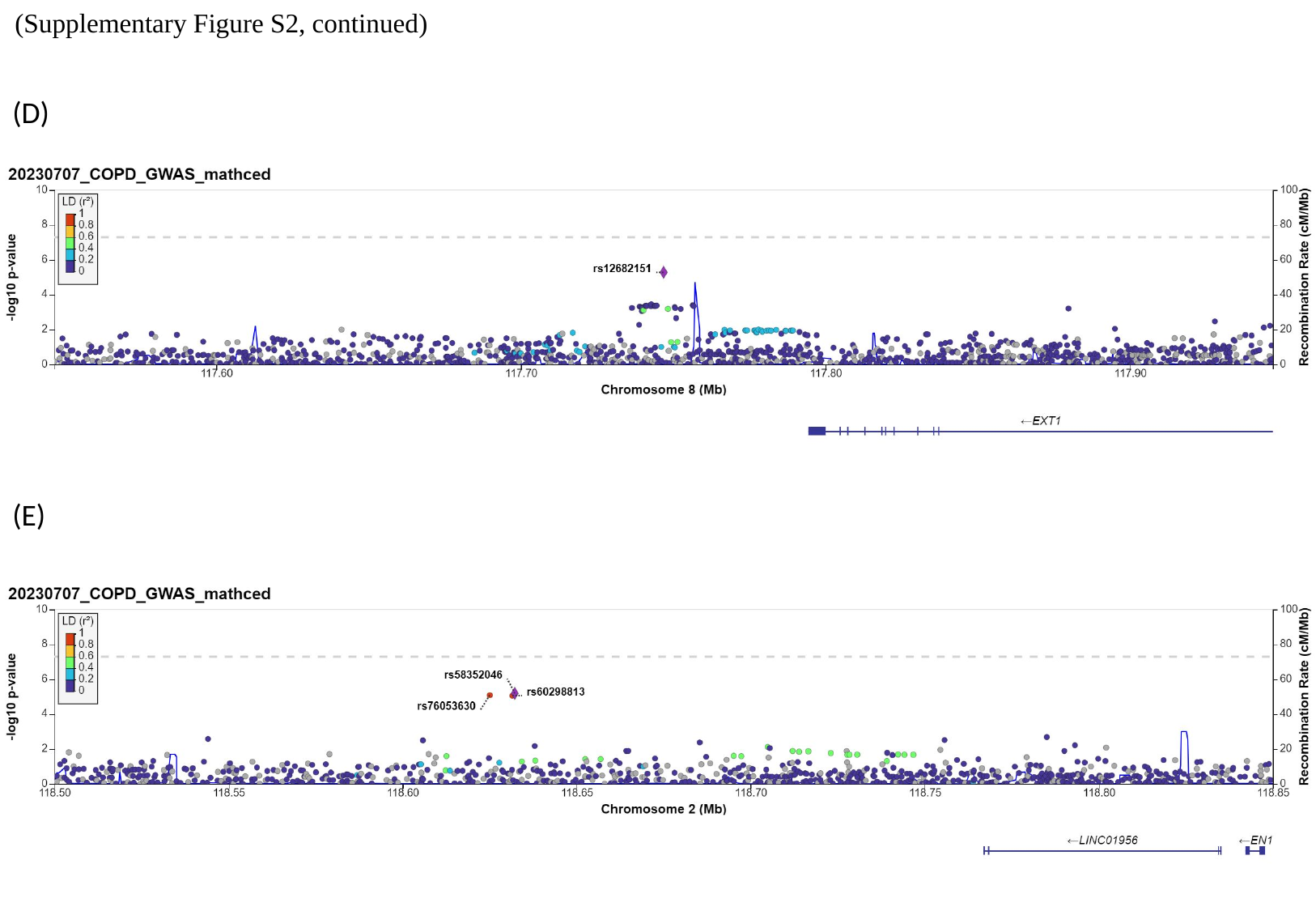

(Supplementary Figure S2, continued)
(D)
(E)

## Slide 4
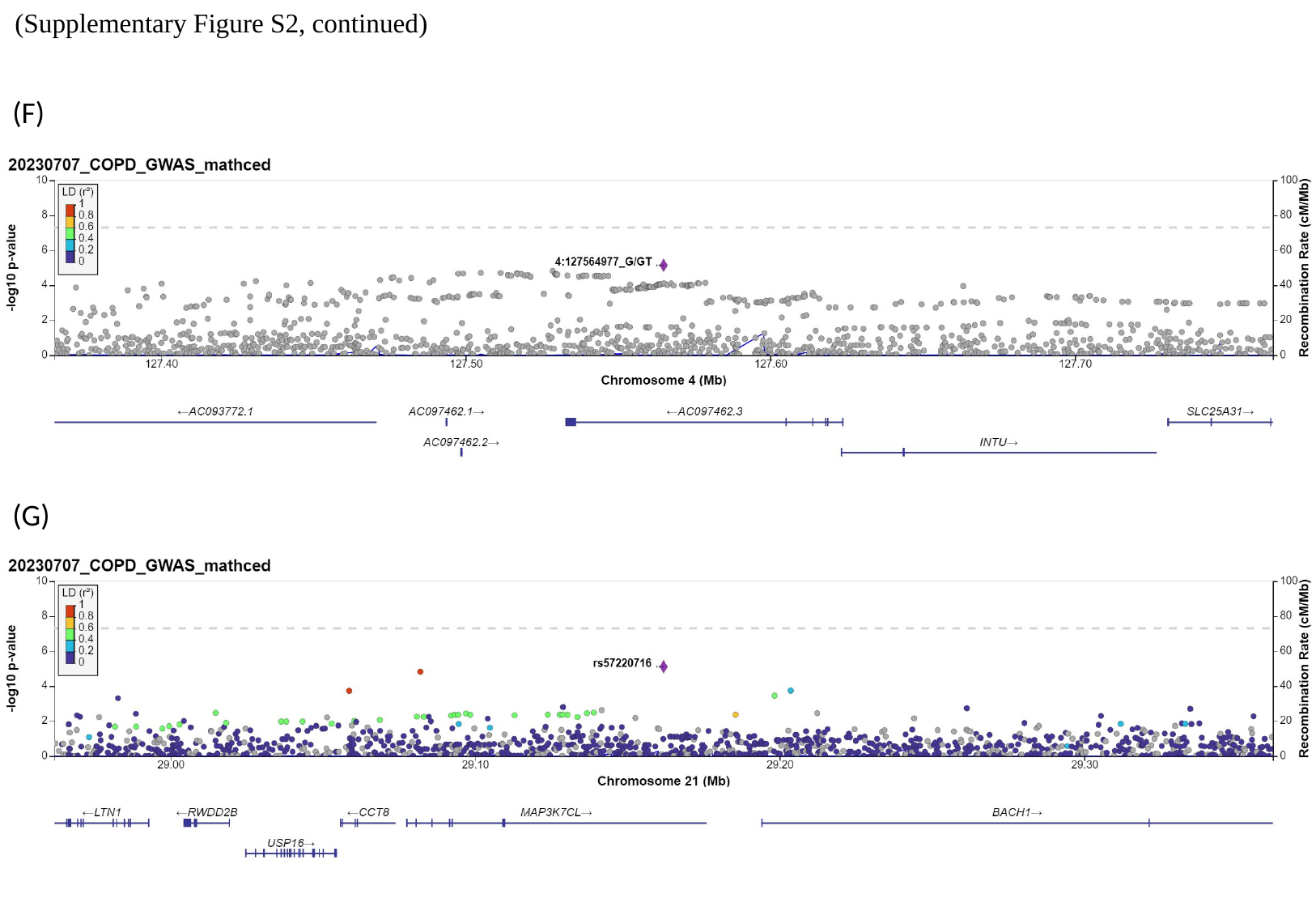

(Supplementary Figure S2, continued)
(F)
(G)

## Slide 5
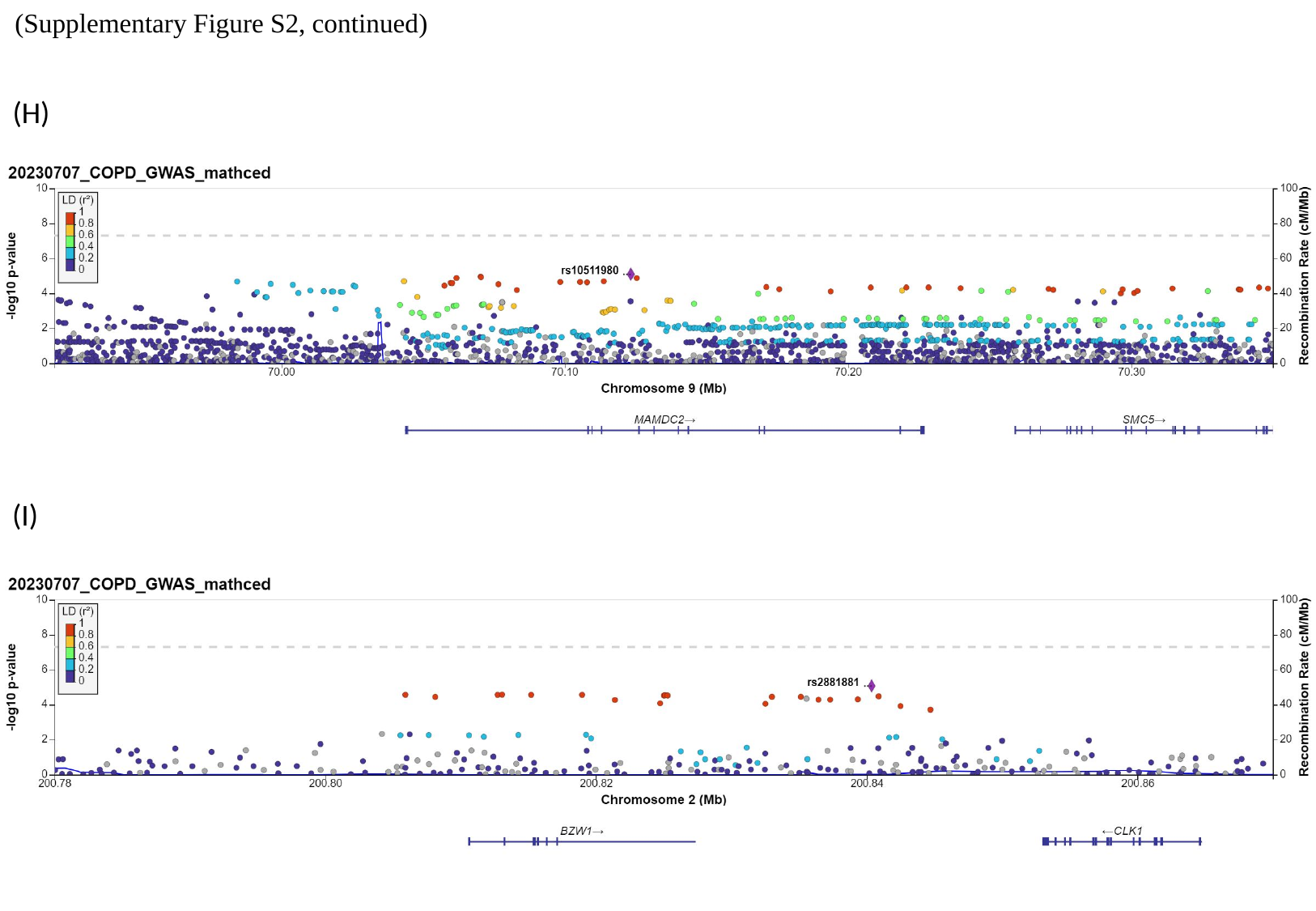

(Supplementary Figure S2, continued)
(H)
(I)

## Slide 6
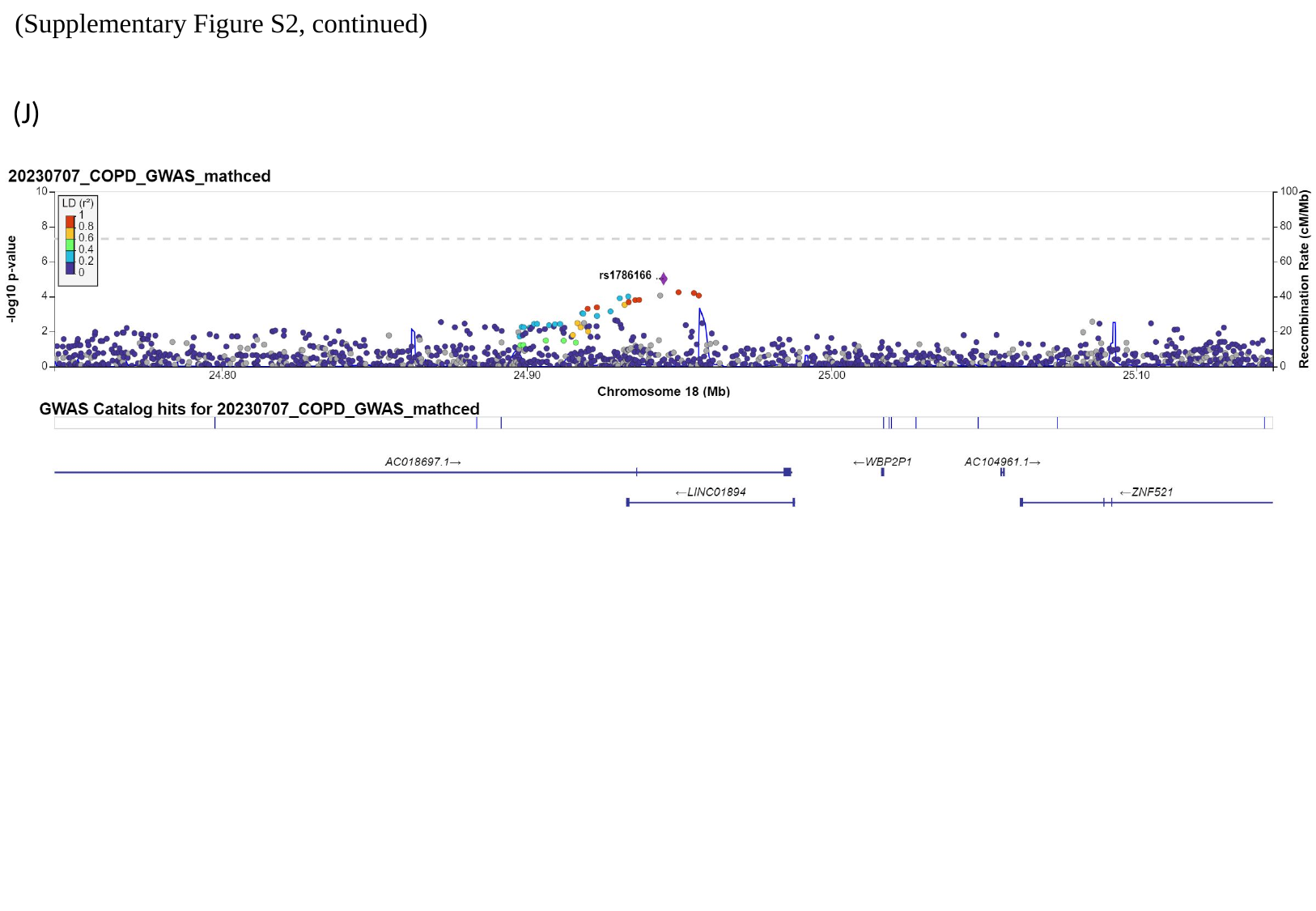

(Supplementary Figure S2, continued)
(J)
