# Supplementary material for: Genome-wide association study identifies novel susceptible loci and evaluation of polygenic risk score for chronic obstructive pulmonary disease in a Taiwanese population
Source: BMC Genomics. 2024 Jun 17;25:607. doi: 10.1186/s12864-024-10526-5 (PMC11184693; doi:10.1186/s12864-024-10526-5)
Supplement: Supplementary file 5 — Supplementary Material 5. [file 12864_2024_10526_MOESM5_ESM.pptx]

## Slide 1
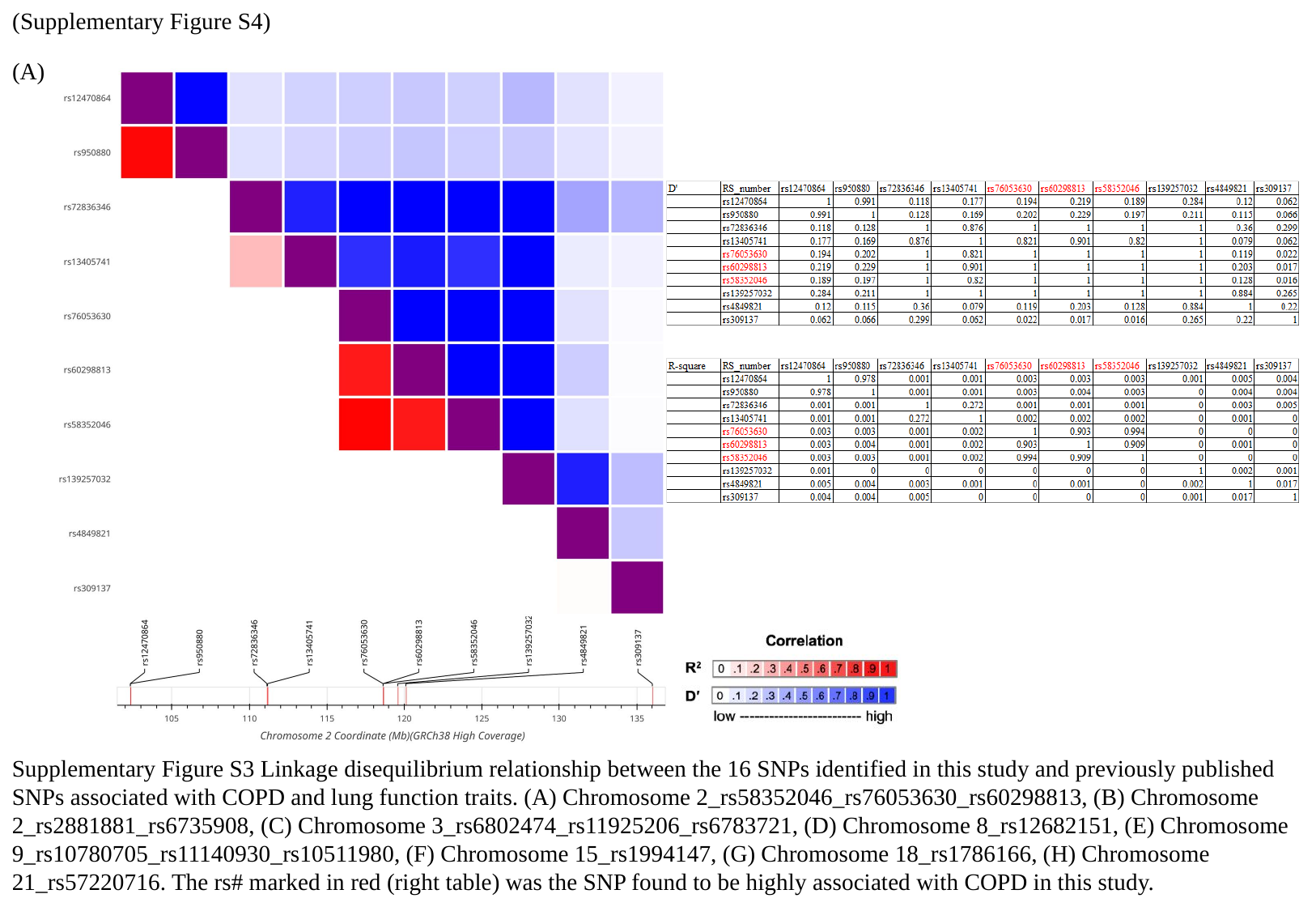

(Supplementary Figure S4)
(A)
Supplementary Figure S3 Linkage disequilibrium relationship between the 16 SNPs identified in this study and previously published SNPs associated with COPD and lung function traits. (A) Chromosome 2_rs58352046_rs76053630_rs60298813, (B) Chromosome 2_rs2881881_rs6735908, (C) Chromosome 3_rs6802474_rs11925206_rs6783721, (D) Chromosome 8_rs12682151, (E) Chromosome 9_rs10780705_rs11140930_rs10511980, (F) Chromosome 15_rs1994147, (G) Chromosome 18_rs1786166, (H) Chromosome 21_rs57220716. The rs# marked in red (right table) was the SNP found to be highly associated with COPD in this study.

## Slide 2
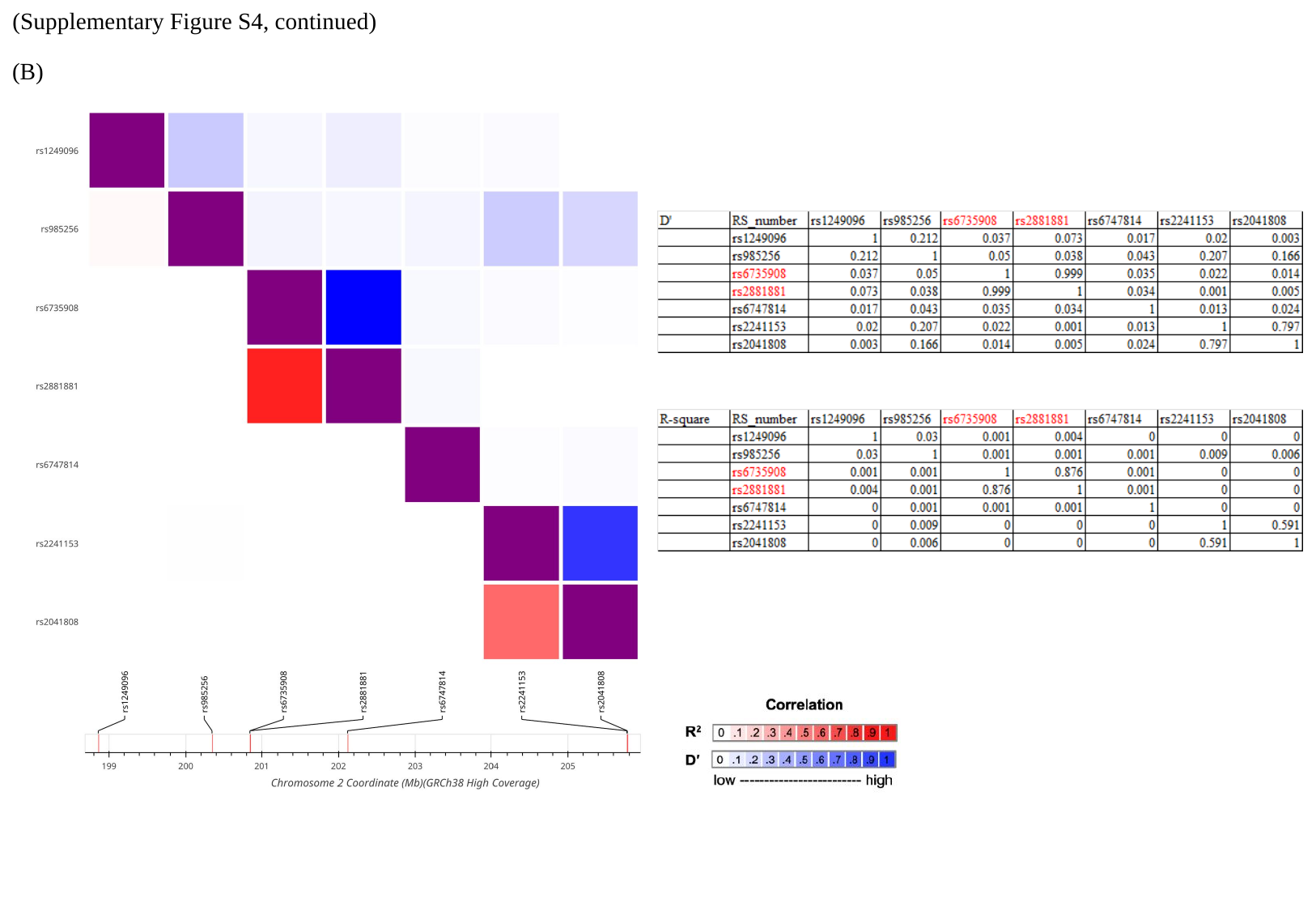

(Supplementary Figure S4, continued)
(B)

## Slide 3
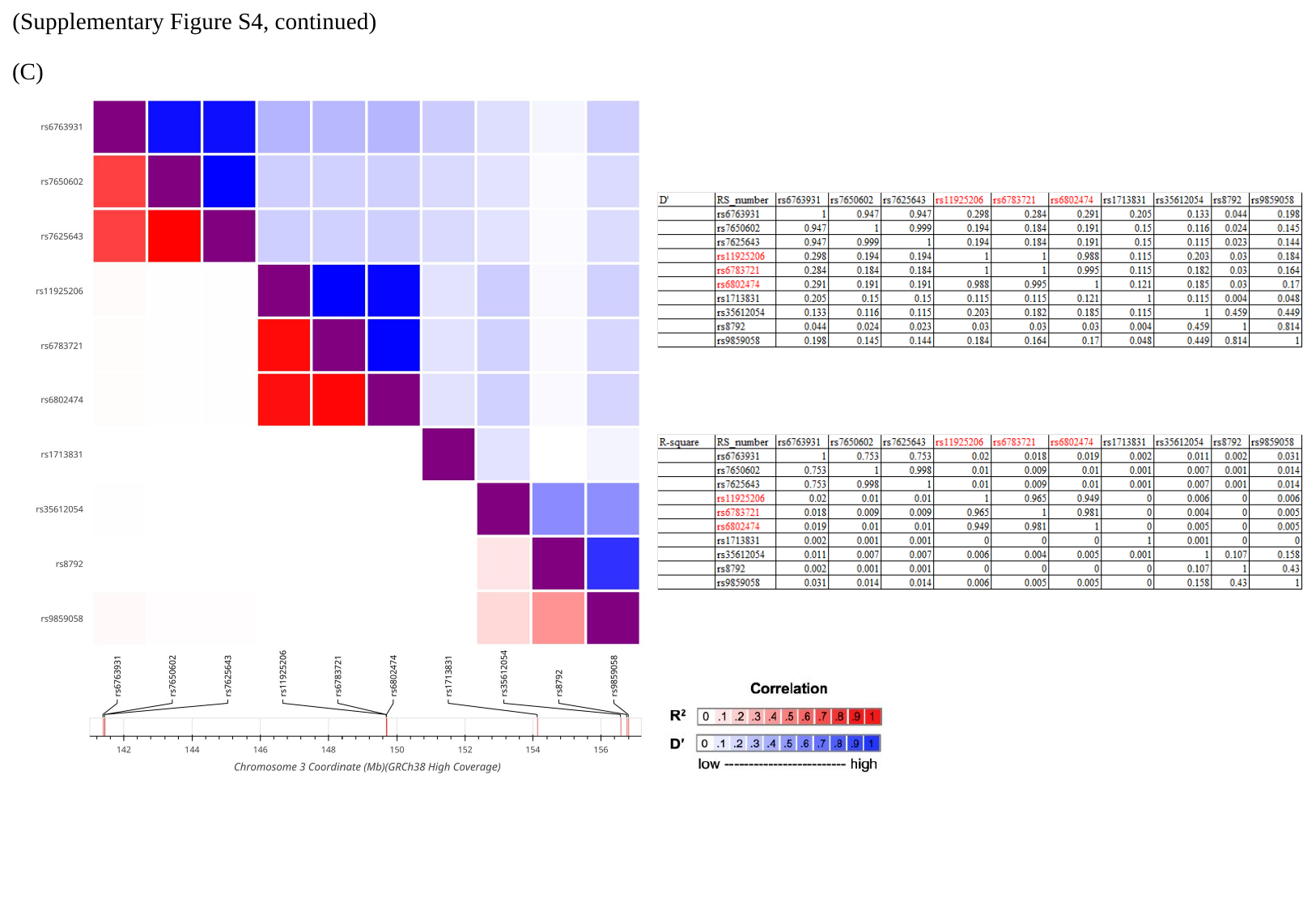

(Supplementary Figure S4, continued)
(C)

## Slide 4
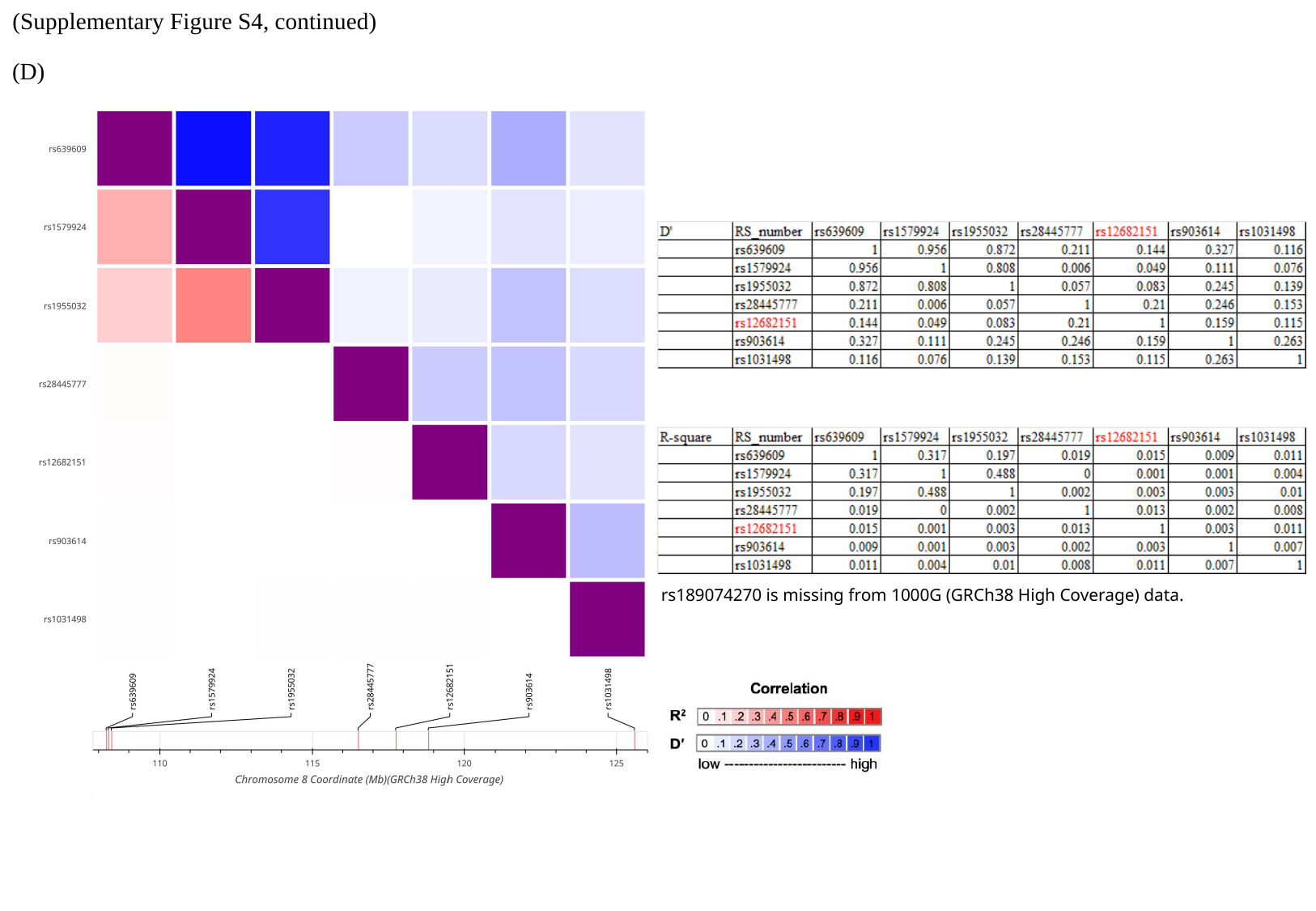

(Supplementary Figure S4, continued)
(D)
 rs189074270 is missing from 1000G (GRCh38 High Coverage) data.

## Slide 5
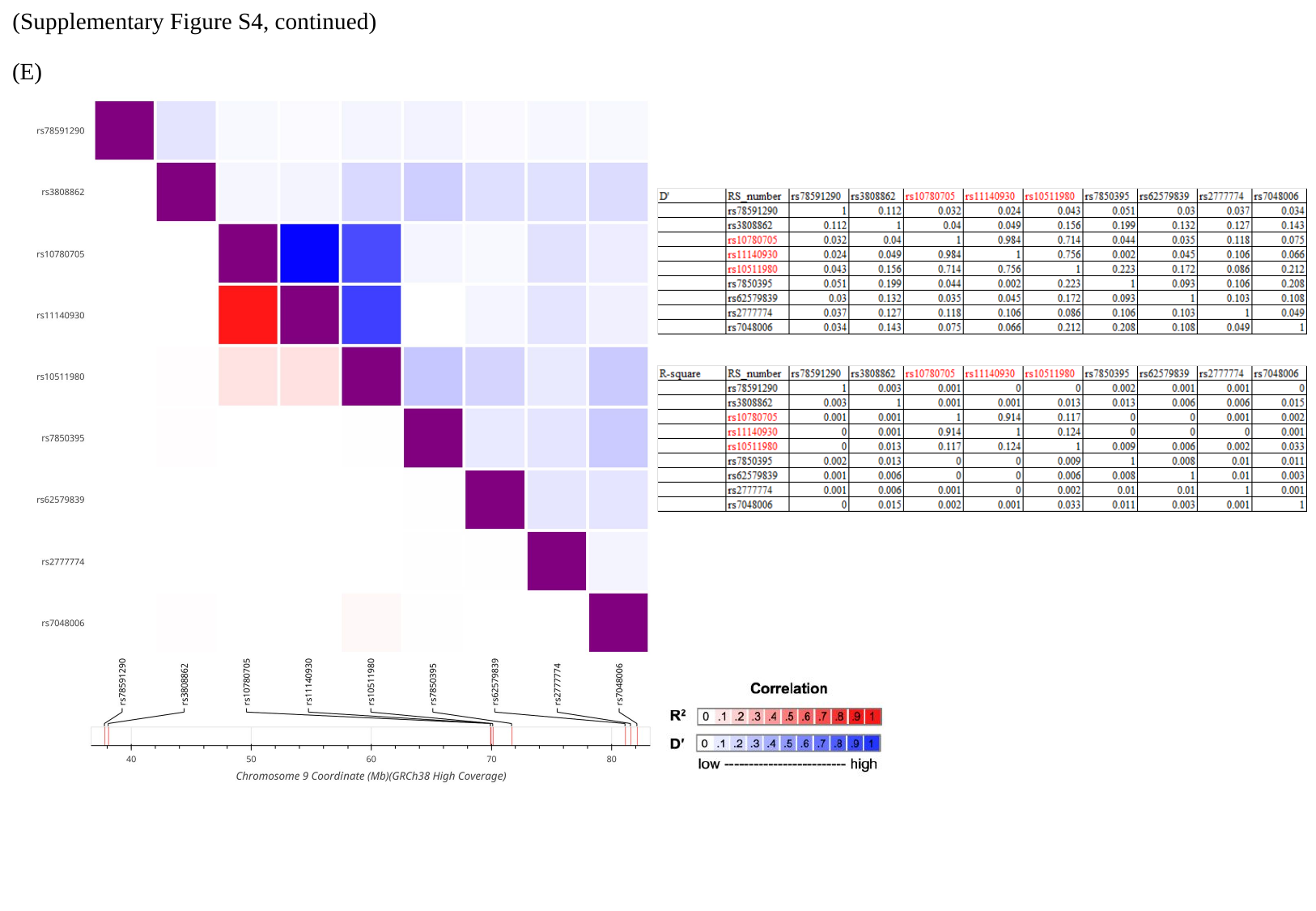

(Supplementary Figure S4, continued)
(E)

## Slide 6
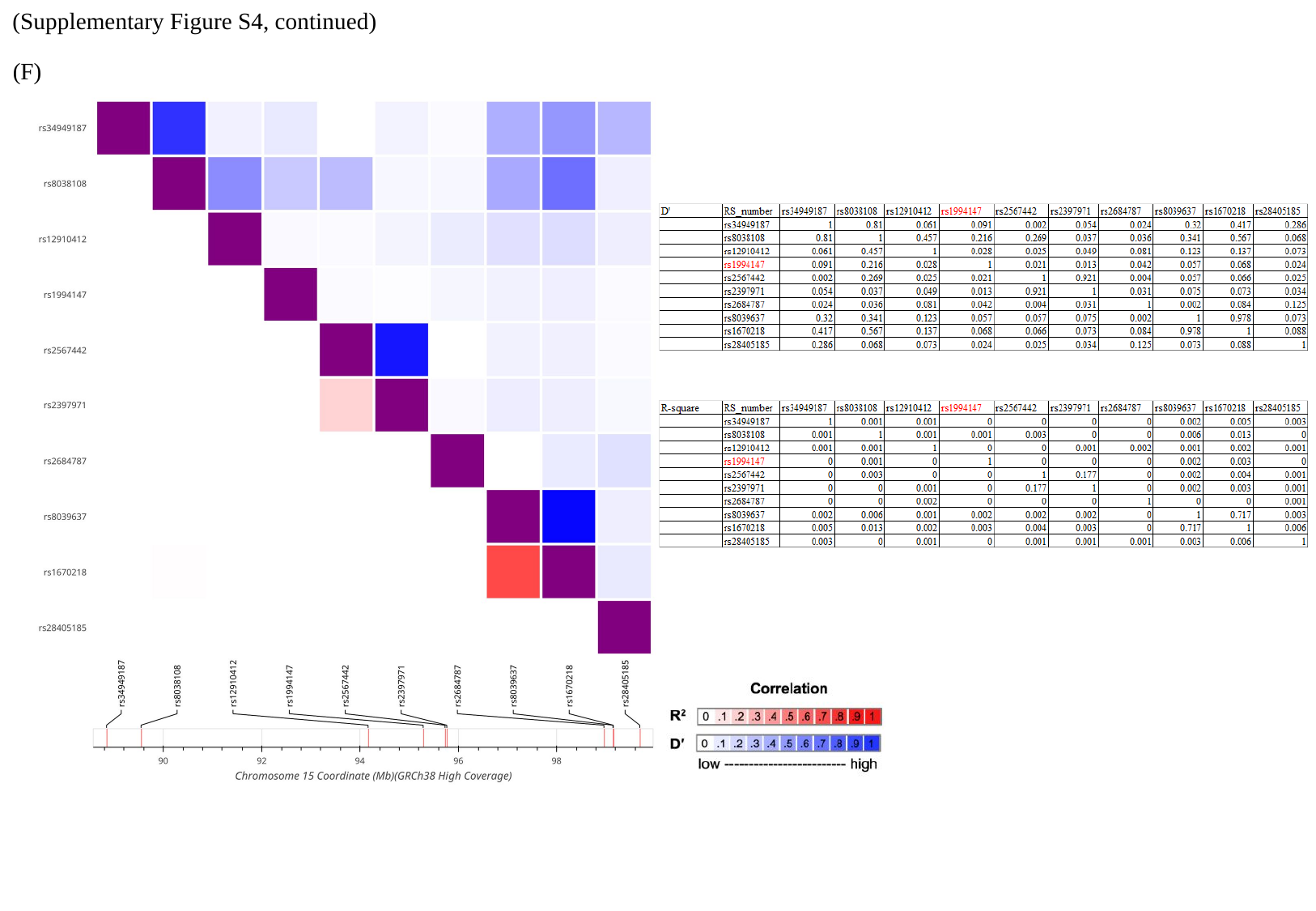

(Supplementary Figure S4, continued)
(F)

## Slide 7
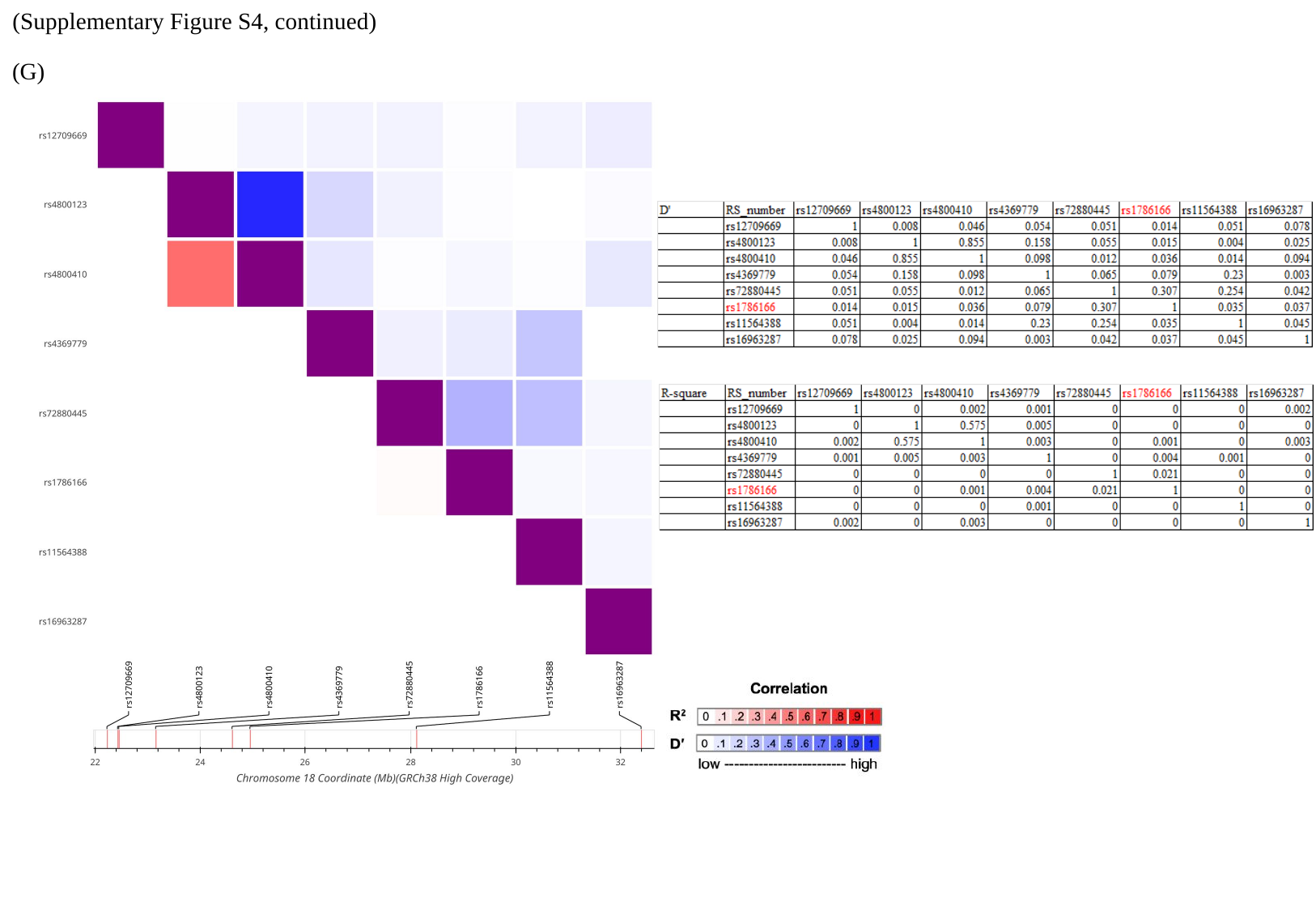

(Supplementary Figure S4, continued)
(G)

## Slide 8
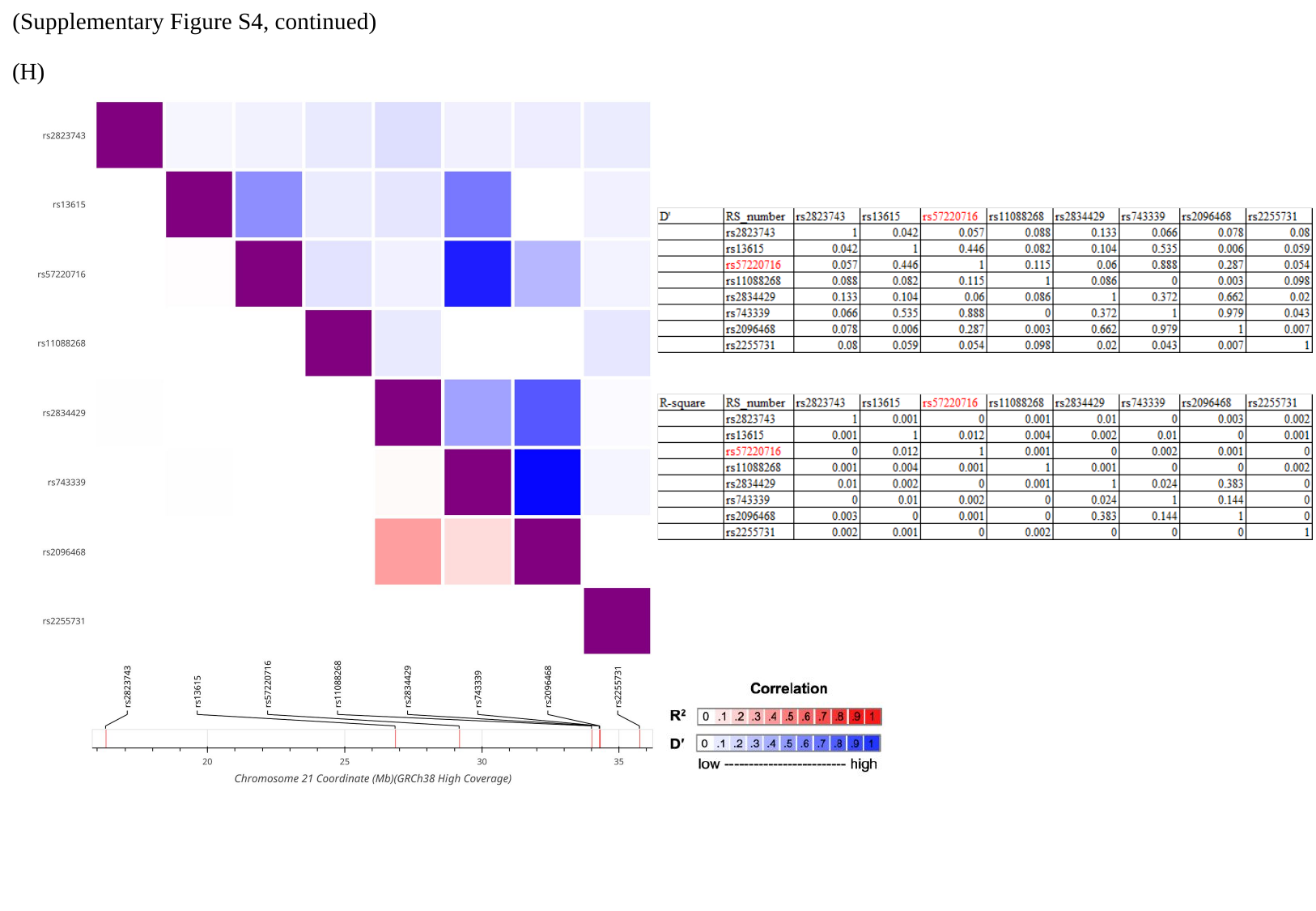

(Supplementary Figure S4, continued)
(H)
