# Supplementary material for: Genome-wide association study identifies novel susceptible loci and evaluation of polygenic risk score for chronic obstructive pulmonary disease in a Taiwanese population
Source: BMC Genomics. 2024 Jun 17;25:607. doi: 10.1186/s12864-024-10526-5 (PMC11184693; doi:10.1186/s12864-024-10526-5)
Supplement: Supplementary file 6 — Supplementary Material 6. [file 12864_2024_10526_MOESM6_ESM.pptx]

## Slide 1
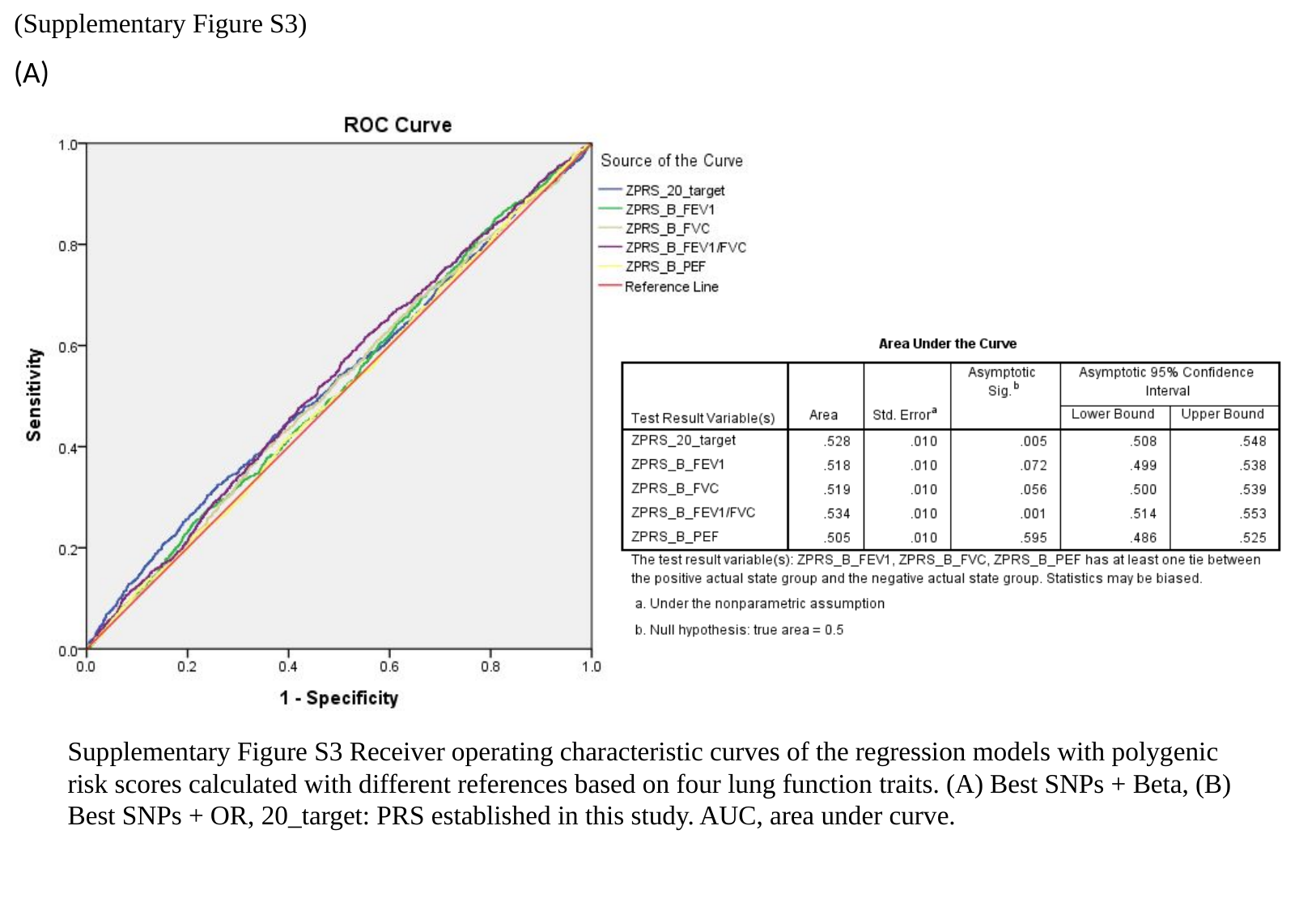

(Supplementary Figure S3)
(A)
Supplementary Figure S3 Receiver operating characteristic curves of the regression models with polygenic risk scores calculated with different references based on four lung function traits. (A) Best SNPs + Beta, (B) Best SNPs + OR, 20_target: PRS established in this study. AUC, area under curve.

## Slide 2
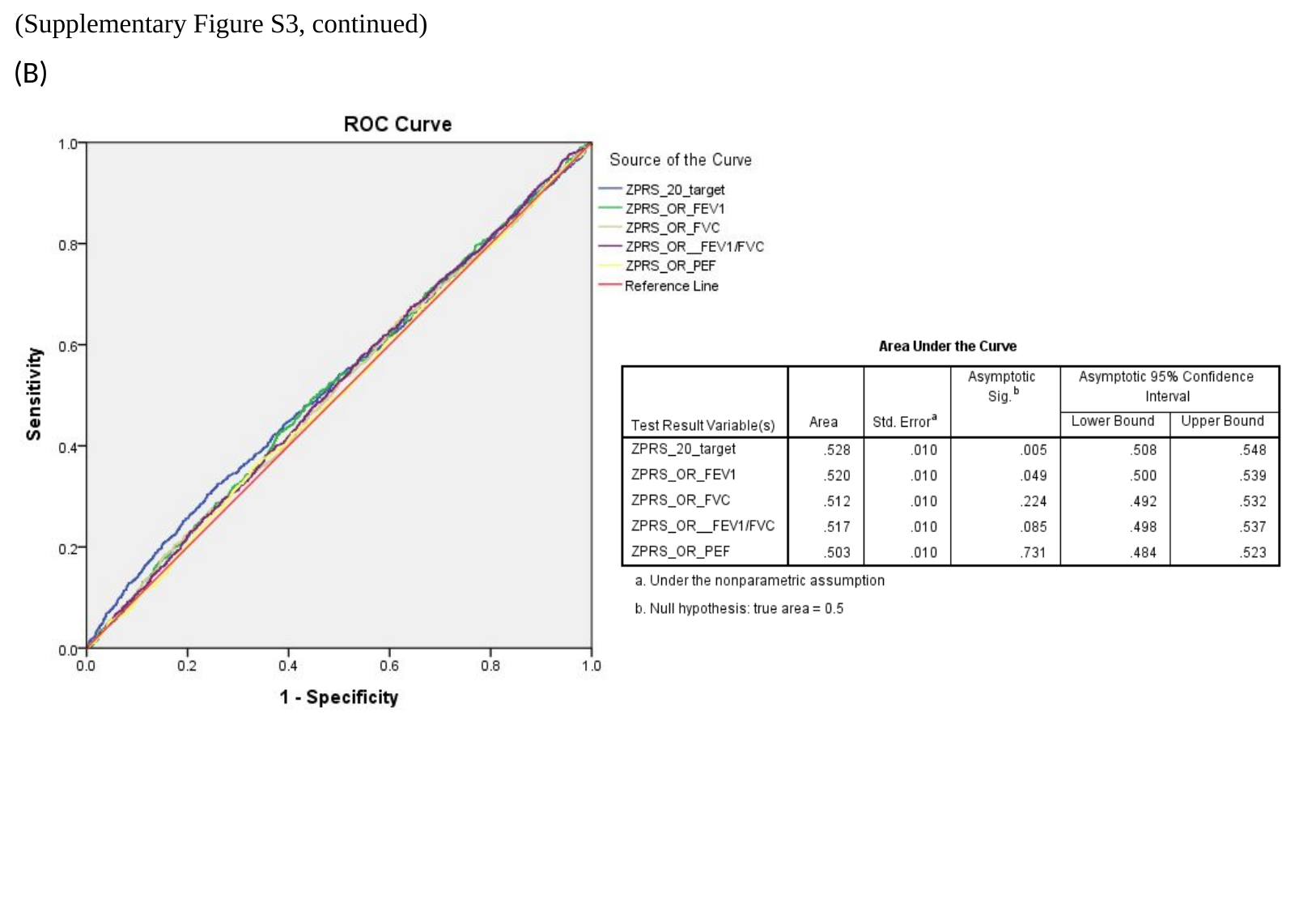

(Supplementary Figure S3, continued)
(B)
